# Mandarin Chinese L1 and L2 complex sentence reading reveals a consistent electrophysiological pattern of highly interactive syntactic and semantic processing: An ERP study

**DOI:** 10.3389/fpsyg.2023.1143062

**Published:** 2023-04-19

**Authors:** Luyao Chen, Mingchuan Yang, Fei Gao, Zhengyuan Fang, Peng Wang, Liping Feng

**Affiliations:** ^1^Max Planck Partner Group, School of International Chinese Language Education, Beijing Normal University, Beijing, China; ^2^Department of Neuropsychology, Max Planck Institute for Human Cognitive and Brain Sciences, Leipzig, Germany; ^3^Institute of Modern Languages and Linguistics, Fudan University, Shanghai, China; ^4^Centre for Cognitive and Brain Sciences, University of Macau, Macao, Macao SAR, China; ^5^Methods and Development Group (MEG and Cortical Networks), Max Planck Institute for Human Cognitive and Brain Sciences, Leipzig, Germany; ^6^Institute of Psychology, University of Greifswald, Greifswald, Germany; ^7^Institute of Psychology, University of Regensburg, Regensburg, Germany

**Keywords:** Chinese complex sentence, syntactic information, semantic information, ELAN, block effect, ERP

## Abstract

**Introduction:**

A hallmark of the human language faculty is processing complex hierarchical syntactic structures across languages. However, for Mandarin Chinese, a language typically dependent on semantic combinations and free of morphosyntactic information, the relationship between syntactic and semantic processing during Chinese complex sentence reading is unclear. From the neuropsychological perspective of bilingual studies, whether second language (L2) learners can develop a consistent pattern of target language (i.e., L2) comprehension regarding the interplay of syntactic and semantic processing, especially when their first language (L1) and L2 are typologically distinct, remains to be determined. In this study, Chinese complex sentences with center-embedded relative clauses were generated. By utilizing the high-time-resolution technique of event-related potentials (ERPs), this study aimed to investigate the processing relationships between syntactic and semantic information during Chinese complex sentence reading in both Chinese L1 speakers and highly proficient L2 learners from South Korea.

**Methods:**

Normal, semantically violated (SEM), and double-violated (containing both semantic and syntactic violations, SEM + SYN) conditions were set with regard to the nonadjacent dependencies of the Chinese complex sentence, and participants were required to judge whether the sentences they read were acceptable.

**Results:**

The ERP results showed that sentences with “SEM + SYN” did not elicit early left anterior negativity (ELAN), a component assumed to signal initial syntactic processing, but evoked larger components in the N400 and P600 windows than those of the “SEM” condition, thus exhibiting a biphasic waveform pattern consistent for both groups and in line with previous studies using simpler Chinese syntactic structures. The only difference between the L1 and L2 groups was that L2 learners presented later latencies of the corresponding ERP components.

**Discussion:**

Taken together, these results do not support the temporal and functional priorities of syntactic processing as identified in morphologically rich languages (e.g., German) and converge on the notion that even for Chinese complex sentence reading, syntactic and semantic processing are highly interactive. This is consistent across L1 speakers and high-proficiency L2 learners with typologically different language backgrounds.

## 1. Introduction

The relationship between syntactic and semantic information during language comprehension has received considerable attention in psycholinguistic academia in recent decades. Whether and to what extent such a relationship is modulated by factors such as language typological differences and language proficiency are not well understood and await specification. Specifically, two research gaps were indentified in the development of research questions of the current study: (1) The relationship between syntactic and semantic processing during Chinese complex sentence reading is unclear; (2) Whether second language (L2) learners can develop a consistent pattern of the target language (i.e., L2) comprehension regarding the interplay of syntactic and semantic processing remains to be determined. Therefore, in *Introduction*, after setting the general background of the ERP (event-related potential) studies in morphologically rich languages and in Mandarin Chinese (1.1 and 1.2), we highlighted the importance of using complex sentences as experimental materials (1.3), which was then followed by the introduction of L2 settings (1.4). At last, we introduced the development of experimental design (esp., the double-violation paradigm) (1.5), and specified the research aims and expectations of the present study (1.6).

### 1.1. A syntax-first model in morphologically rich languages

A prominent cognitive model of auditory language comprehension (Friederici, 2002, 2011, 2017) holds that syntactic information precedes and then interacts with semantics, and this early syntactic information mainly deals with syntactic categories of words. In particular, word category information has been recognized as the foundation of syntactic structure building (Lenneberg, 1967; Chomsky, 1995; Hornstein and Nunes, 2008; Hornstein, 2009; Hornstein and Pietroski, 2009; Adger, 2013; Miyagawa et al., 2013; Epstein et al., 2014; Hoshi, 2018, 2019), inspiring a series of studies on syntactic processing that adopt various word category information (Bemis and Pylkkänen, 2011; Miyagawa et al., 2013; Fujita, 2014; Goucha and Friederici, 2015; Friederici, 2017; Goucha et al., 2017; Zaccarella et al., 2017; Chen et al., 2019). Using the event-related potentials (ERPs) technique of high temporal resolution, previous studies have investigated the interplay between syntactic category and semantic information processing in morphologically rich languages (especially German). The evidence converged to demonstrate a priority of syntactic processing over semantic processing, supporting a “syntax-first model.” For instance, in the double-violation/combined violation paradigm (e.g., Hahne and Jescheniak, 2001; Hahne and Friederici, 2002) during German sentence comprehension, the simultaneous violation of syntactic and semantic information elicited pronounced early left anterior negativity (ELAN) in the time window of 120–200 ms (see Neville et al., 1991, Friederici et al., 1993 for more information on ELAN; see Friederici and Weissenborn, 2007 for a systematic review), a negative component assumed to signal initial syntactic processing, while N400 was absent, a classic negative component reflecting semantic violations in the time window of 300–500 ms (see Kutas and Hillyard, 1980, 1983, and Friederici et al., 1993 for more information on N400; see Kutas and Federmeier, 2011 for an overview). ELAN could therefore index a temporal priority of syntactic category processing. The absence of N400 suggested an inhibition on the following semantic processing from the failure of syntactic encoding, which was defined as a “block effect” (Yu and Zhang, 2008). Experiments on French (Isel et al., 2007), English (Yamada and Neville, 2007), and Dutch (Hagoort et al., 2003) reported similar electrophysiological patterns regarding the time course of syntactic processing in sentence comprehension. At a two-word-phrase level, syntactic violation in German could elicit an ELAN-like early syntactic negativity (Maran et al., 2022). Therefore, syntactic processing in such languages was primarily evidenced to be both temporally and functionally prior to semantic processing. Nevertheless, emerging evidence from Indo-European languages began to conflict with the syntax-first account. For example, in a recent ERP study of French sentence reading where uninflected nouns and verbs were swapped (Fromont et al., 2020), ELAN did not appear in syntactic category violations. These findings suggested that inflectional cues might trigger and thus speed up syntactic processes (see also Dikker et al., 2009).

### 1.2. The interplay of syntactic and semantic processing in Mandarin Chinese

Syntactic category information is primarily marked by morphological inflections in these morphologically rich languages. For instance, “*gegessen*” (eaten) is the past participle of “*essen*” (eat) in the German material “*Das Brot wurde gegessen*” (The bread was eaten) in Hahne and Friederici’s study (2002). These morphological cues might facilitate access to word category information and lead to syntactic priority (Friederici, 2017). However, there is no such inflection in morpho-syllabic languages such as Mandarin Chinese (hereafter, Chinese) (DeFrancis, 1989; Shen, 2016; Gao et al., 2022), where word order and functional words constitute the principle grammatical operations (Zhu, 1982). Existing studies have therefore examined whether the syntax-first model fits Chinese processing as well by using various syntactic structures. Ye et al. (2006) first approached this issue in the Chinese BA (“*把*”)^1^ structure (i.e., subject + BA + object + VP). Their results revealed that double-violations elicited ELAN and a continuous negative wave in the time window of 250–400 ms. This pattern was similar to the findings of morphologically rich languages. However, this study could not rule out the possibility that N400 was included in the continuous negative component due to the existence of numerous homonyms in the Chinese lexicon (Zhang et al., 2010). Yu and Zhang (2008) also examined this issue in the Chinese BA structure with more careful control and included semantic violations and double violations. The reason for not including syntactic violations was twofold. First, Chinese word category violations are always accompanied by semantic violations such that there should be no pure syntactic violations in Chinese. Second, to examine whether syntactic processing would manifiest a temporal and functional priority in sentence comprehension, the presence of ELAN and “block effect” in the double-violation condition is sufficient to draw a conclusion. Interestingly, their results demonstrated a biphasic N400-P600 pattern, where double violations were associated with significantly greater N400 and P600 compared to semantic violations. In light of the null results that both ELAN and the block effect did not appear, the authors concluded that Chinese syntactic processing might not exhibit a priority over semantic operations during sentence comprehension, and the two types of processing should be highly interactive. These results were replicated in a study focusing on BA construction and subject-verb-object (SVO) structures by Zhang et al. (2010). In addition, Wang S. et al. (2013) and Wang et al. (2015) manipulated the transitivity of verbs in Chinese BA/BEI (“*被*”)^2^ and NP1 + VP + NP2 structures in light of a double-violation paradigm. Their results revealed that double violations and pure semantic violation elicited comparable N400 and late positivities, again not supporting the application of the syntax-first model to Chinese sentence comprehension. Two additional studies re-examined this issue in Chinese passive BEI (Zeng et al., 2020) and Qing (“*请*”)^3^ structures (Yang et al., 2021). Zeng et al. (2020) obtained similar results as Zhang et al. (2010), Wang S. et al. (2013), and Wang et al. (2015), while Yang et al. (2021) also observed an interaction between syntactic category and semantic processing in an early time window of 100–300 ms. A very recent study using intracranial high-density electrocorticography found that syntactic and semantic processing in Chinese showed spatial–temporal separations (Zhu et al., 2022). Only the local syntactic violation, not the syntactic category violation, elicited an ELAN-like component in the early time window. However, it is not clear whether this inconsistent pattern resulted from the different types of syntactic violations or from the differences in local preferences (see below).

### 1.3. Local versus long-distance dependencies: Limitations on syntactically simple sentences

Although the aforementioned studies were in favor of the null primacy of syntactic information of distinguishing structures in Chinese simple sentences, it remains unclear how syntactic and semantic processing interact with each other when reading relatively complex sentences. One significant feature of the human language faculty is that language parsers can comprehend complex hierarchical sentences containing center-embedded relative clauses (Makuuchi et al., 2009, 2013; Friederici, 2017). For example, in the complex sentence “The dog the cat chased barked,” the relative clause “the cat chased” is embedded between the subject “the dog” and the verb “barked,” which requires processing on the long-distance/nonadjacent dependency. This hierarchical syntactic structure reflects the complexity of human capacities of sequence processing (Petkov and ten Cate, 2020; Wilson et al., 2020). In contrast, simple sentence processing implicates mental operations on local dependency. For example, in the double-violation condition “*警察交战骗局*…” (“police fought the fraud”) (Wang et al., 2015), the verb “*交战*” (fight) and the object “*骗局*” (fraud) are locally adjacent. Such local dependencies depict the adjacent collocation between word categories, which could be confounded with the more cognitive-general effect of “local preference” in syntactic processing. Specifically, local preference originates from the limitation of human cognitive resources, such that language users tend to integrate syntactically local or adjacent information as early as possible (Gibson, 1998). As such, the ERP components associated with local syntactic violations (e.g., ELAN) could indicate both word category violations and local preference violations. Moreover, a local syntactic violation may also be mixed with a violation of template matching, resulting in an unclear detection of the violation *via* the failure to build up a grammatical phrase (e.g., “the in,” a determiner cannot be combined with a preposition to form a determiner phrase) or a mismatch with an *a priori* template (e.g., “the in” does not match the “determiner noun” template) (Friederici, 2017; Goucha et al., 2017). Therefore, the existence of local syntactic relations in simple sentences fails to provide optimal material for examining the interplay between syntactic and semantic processing. Examining this issue in complex sentences might overcome these limits and substantially advance our understanding of the interaction between these types of information as well as the human language faculty (Hauser et al., 2002).

In particular, syntactic complexity could be measured by integration and storage costs, instead of the mere sentence length (Gibson, 1998; Chesi and Moro, 2015). In specific, integration cost is concerned with the process of integrating syntactic categories, while storage cost is qualified by the number of the involved categories. Both integration and storage cost are impacted by locality. As such, sentences containing center-embedded subject relative clauses manifest great complexity and processing difficulties in light of their integration and storage cost, which also highlights the crucial role of word category information. Chinese complex sentences containing center-embedded subject relative clauses, such as “*警察抓了偷电脑的小偷*” (“Police caught the thief who stole a computer”), in which “*小偷*” is non-adjacently dependent with the main verb “*抓*” as its object and with the verb “*偷*” in the relative clause as the subject, implicate even higher complexities and more processing difficulties (Hsiao and Gibson, 2003; Chen et al., 2008; Chen and Ning, 2008; Zhang and Yang, 2010; Zhou et al., 2010; Liu, 2011; Liu et al., 2011; Wang, 2011; He et al., 2012; Feng and Wang, 2013; Wang and Bing, 2013; Yan, 2018; Xu et al., 2019; Zhou et al., 2020). The current study therefore adopted these complex sentences with subject relative clauses center-embedded as experimental materials to highlight the interactive relationships between syntactic category information and semantics in Chinese sentence reading.

### 1.4. The interplay of syntactic and semantic processing in L2 learners

In addition to the language typological distinctions in the interplay between syntactic and semantic information, as manifested by the distinct patterns between morphologically rich Indo-European languages and morpho-syllabic languages, language proficiency might be another critical factor that affects the relationships between syntactic and semantic processes during language comprehension (Kotz, 2009). Zhang et al. (2010) concluded that language experience might affect the interplay between syntactic and semantic processes. A recent review (Niharika and Prema Rao, 2020) also proposed that language typological differences are closely associated with the language-specific brain correlates underlying syntactic processing. However, it is largely unknown to what extent language experience or background modulates this pattern in second language (L2) settings. For example, given adequate language exposure, do L2 learners employ the processing strategies from their first language (L1) or, alternatively, do they resemble native speakers of the target language? This issue would be more intriguing if learners’ L1 and L2 manifested marked differences regarding linguistic typology.

More importantly, since the ability to encode syntactic information during complex sentence comprehension constitutes a crucial part of the human language capacity, it is critical to know whether and how L2 learners can acquire the native-like strategies of discerning syntactic and semantic information at the neuroscientific level. Specifically, the unified competition model (MacWhinney, 1997, 2005) proposes that L2 learners employ the cognitive resources and processing strategies from their L1 to deal with L1-L2 shared structures. In addition, given presumably adequate L2 proficiency, learners could develop native-like sensitivity to syntactic information and eventually achieve native-like attainment (following the convergence hypothesis, e.g., Steinhauer et al., 2009). Fromont et al. (2020) compared the ERP responses to L2 French sentences containing syntactic-category violations of native English speakers (with an intermediate level in L2 French) with those of native French speakers. Interestingly, L1 speakers and L2 learners showed differing electrophysiological patterns such that the L2 group only manifested an N400 effect in syntactic violations, while the L1 group displayed a biphasic N400-P600 effect.

However, few studies have examined whether L2 learners could develop the processing patterns of the target language regarding the interplay of syntactic and semantic information across typologically distinct languages. Moreover, there have been few investigations into the interplay between syntactic and semantic information among highly proficient L2 learners. By using comparatively simple structures (i.e., BEI structure), Yang (2012) found results supportive of an interactive model over a syntax-first model in Chinese comprehension for both Chinese L1 speakers and Chinese L2 learners (German L1 speakers). However, this study failed to make a direct comparison between the two groups. Additionally, it remains unclear whether this pattern extends to complex hierarchical sentences. Going beyond this, our study aimed to include high-proficiency Chinese L2 learners whose native language was Korean to compare their neurocognitive patterns with those of Chinese native speakers during complex sentence reading. As an agglutinative language, Korean morphology includes abundant word form changes to mark syntactic features whose diversity lies between German and Chinese (Zhang et al., 2011). For instance, Korean relative clauses are usually led by verb form changes, while Chinese relies on the functional word “*的*” (de, meaning: of) (Zhao, 2011). Specifically, employing Chinese L2 learners whose native languages are morphologically rich could effectively eliminate interference from their L1 processing strategies (e.g., semantic analysis preference). Furthermore, the inclusion of L2 learners of high proficiency could rule out the confounding effect of language incompetence. Through these manipulations, we aimed to provide critical insights into the interplay between syntactic and semantic information during Chinese complex sentence comprehension, thus drawing on a comparison of L1 versus L2.

### 1.5. Development of the experimental design

With regard to the experimental design, double-violation can be realized by altering either the sentential context (e.g., Hahne and Friederici, 2002; Frisch et al., 2004; Ye et al., 2006; Zhang et al., 2010; Wang X. et al., 2013) or the critical word (Yu and Zhang, 2008; Zhang et al., 2010, 2013; Wang S. et al., 2013; Lu, 2015; Wang et al., 2015; Yang et al., 2015; Li, 2016; Fromont et al., 2020). Sentential context alternation might elicit imbalanced ERP effects on the same critical word. For instance, Hahne and Friederici (2002) kept the critical word (e.g., *gegessen*, “eaten”) unchanged across three violation conditions (i.e., double violations, semantic violation, and syntactic violation). The critical word was embedded in a “Verb + Preposition + Noun” structure in the double-violation condition but in a “Verb + Noun” structure in the other two conditions. Ye et al. (2006) placed the semantic violation after the “BA + Noun” construction, while syntactic violation and double violations appeared immediately after the preposition “BA.” These contextual asymmetries might result in the so-called “spillover effect” on ERP signals, which would contaminate the ERP data locked to the critical words (Steinhauer and Drury, 2012; Zhang et al., 2013; Fromont et al., 2020). As the current study focused on the complex structure of Chinese sentences, we needed to keep the sentential contexts unchanged across conditions. To better evaluate the ERPs on critical words and avoid the spillover effect, this study manipulated violations by altering critical words.

The critical word alteration should take the processing differences of different word categories (e.g., Ns and *Vs*) into account. In particular, related Chinese studies have attempted to change the critical words from verbs to nouns in double violations (e.g., Yu and Zhang, 2008; Zhang et al., 2010, 2013; Lu, 2015; Yang et al., 2015; Li, 2016), which might involve the confounding effect of word category processing. For example, Ma et al. (2007) reported that Chinese nouns could elicit greater N400 amplitudes than Chinese verbs. In that case, we could not rule out the possibility that the greater negativities observed in these studies could stem from word category processing *per se* rather than semantic and syntactic violations. To resolve this confounder, Yu and Zhang (2008) performed a *post hoc* analysis comparing the negativity distribution of double violations on the scalp with that of word category processing, which revealed that ERP results were not contaminated by the word category processing difference. However, the scalp distributions of word category processing were retrieved from previous studies, which might not match the case of their critical words. Alternatively, some studies have included control sentences containing the critical words (Steinhauer and Drury, 2012; Fromont et al., 2020) as a reference to better evaluate the effects of syntactic and semantic violations. For example, Fromont et al. (2020) manipulated the prior context of the critical words while altering the critical words during French sentence comprehension. Unfortunately, this manipulation does not fit Chinese complex sentences, in which it is difficult to ensure that the experimental and control sentences are comparable. It is even more difficult to change the prior context of the critical words while keeping the structure unchanged in Chinese given the language typological difference between French and Chinese.

To resolve these limitations, the current study tried to eliminate the word category difference by two approaches. First, we altered the critical nouns in double violations into verbs instead of the other way around. In this case, the observed greater negativities elicited by double violations during the time-window of N400 than the other conditions (if any) would be more convincing for the existence of semantic processing. Additionally, we included a single-word reading session in which participants read identical critical words as the sentence comprehension session. By subtracting the ERP signals locked to the single words from those locked to the critical words in the experimental sentences, we hoped to better eliminate the confounding effects of word category processing differences and other physical properties of no interest.

### 1.6. Research aims and expectations

By tentatively developing the critical word alternation design by subtracting the ERP signals of processing single words *per se* from those of the same critical words, the current study aimed to examine the interplay between syntactic and semantic processing among Chinese native speakers and highly proficient L2 learners from a distant linguistic background when reading Chinese complex sentences containing center-embedded relative clauses, which should be highly dependent on word-category-based syntactic processes in linguistic theories. In particular, we are interested in whether Chinese complex sentences manifest a syntax-first pattern, which would be shown by the ELAN and the block effect. Crucially, we wanted to investigate whether Chinese L2 learners would acquire syntactic and semantic processing strategies at a relatively higher proficiency with regard to the interplay of syntactic and semantic processing during the ELAN, N400, and P600 time windows. If highly proficient Chinese L2 learners could exhibit a similar ERP pattern to that of L1 speakers, we could suggest that L2 proficiency might fill the gap in language typological differences to tune L2 reading performance.

## 2. Methods

### 2.1. Participants

A total of 42 adults were recruited, including 21 Mandarin-Chinese native speakers (6 males, 23.48 ± 2.91 years) and 21 highly proficient Chinese L2 learners whose native language was Korean (7 males, 23.29 ± 3.51 years). The sample size was consistent with existing related ERP studies (e.g., Yang, 2012; Wang S. et al., 2013; Wang et al., 2015). Specifically, the Chinese L2 learners from South Korea were year-four college students or postgraduates majoring in Chinese, all of whom passed the HSK (i.e., Hanyu Shuiping Kaoshi, a standardized Chinese proficiency test, ranging from bands 1 to 6) with band 5 or above and were verified as highly proficient learners by their instructors. All participants were right-handed with normal or corrected-to-normal vision and reported no reading difficulty. They all signed the consent form prior to the experiment and received a monetary reward afterward. This study was approved by the Ethics Committee of Beijing Normal University. Data from one L1 speaker and one L2 learner were excluded from the analyses due to excessive ERP artifacts, such as blinks.

Given the long-distance dependency between critical words and other related words in the experimental materials and the word-by-word presentation format in the current study, we needed to exclude the confounding effect from working memory capacity differences between the two groups. The working memory capacity measure was adapted from the automated operation span task by Unsworth et al. (2005). In this task, participants were asked to first judge the correctness of a hybrid math operation [e.g., (8–2) × 3 = 18?] and then to memorize a random English letter following this operation. As the number of operations increased, the participants needed to remember more letters. At the end of each trial, they were required to recall all the letters in each trial in the order of their appearance. An independent-sample t test showed that L1 speakers [*N* = 20, ACC (accuracy) = 64.15% ± 6.78%] and L2 learners (*N* = 20, ACC = 61.55% ± 7.14%) yielded no significant group difference in working memory capacity, *t*(38) = −1.18, *p* = 0.245.

### 2.2. Materials

There were 120 experimental sentences in total, with 40 in each condition (NORM: semantically and syntactically normal sentences, SEM: sentence with semantic violation, and SEM + SYN: sentence with double syntactic-semantic violations; see Table 1 for examples). In addition, to counterbalance the number of correct and violated sentences, 40 filler sentences were included (see also Wang et al., 2015). Each experimental sentence contained a center-embedded relative clause, such as “*小张拿着切水果的小刀过来了*。.” In the SEM condition “*小张拿着切水果的钢琴过来了*。,” even though “*钢琴*” (piano) is a noun without syntactic violation, the sentence is semantically violated because “piano” could never be used to “cut the fruit.” In addition, in the SEM + SYN condition, “*举办*” (hold) is a verb resulting in both syntactic and semantic violations because “hold” could neither “cut the fruit” nor “be taken.” The filler sentences were similar to the experimental sentences in length but did not contain complex relative clauses, such as “*小张拿走了孩子的玩具很高兴*.” The rationale of designing such filler sentences is as following. In light of existing studies (e.g., Wang S. et al., 2013; Wang et al., 2015), the fillers should meet the following criteria. First, the purpose of filler was to balance the correct and incorrect sentences. Second, to avoid the participants’ strategic responses, the fillers should be comparable with the experimental sentences regarding the superficial linguistic features (e.g., the number of nouns/verbs, sentence length). Thus, the filler sentence structure and the complex sentence structure in the present study share a similar syntactic frame: “N + V + V + N + de + N…” The correspondence of the overall structure between fillers and experimental sentences was not obligatory.

**Table 1:** Examples of experimental conditions and fillers.

Condition	Example sentence
NORM	小张 | 拿着 | 切 | 水果 | 的 |**小刀** | 过来了。 Zhang | was taking | cut | fruit | de (*functional word*) |**knife** | came over. (Zhang was coming over with the **knife** which is used to cut fruits.)
SEM	小张 | 拿着 | 切 | 水果 | 的 |**钢琴** | 过来了。 Zhang | was taking | cut | fruit | de (*functional word*) |**piano** | came over. (Zhang was coming over with the **piano** which is used to cut fruits.)
SEM + SYN	小张 | 拿着 | 切 | 水果 | 的 |**举办** | 过来了。 Zhang | was taking | cut | fruit | de (*functional word*) |**hold** | came over. (Zhang was coming over with the **hold** which is used to cut fruits.)
Filler	小张 | 拿 | 走了 | 孩子 | 的 |**玩具** | 很高兴。 Zhang | took | away | children | de (*functional word*) |**toy** | was very happy. (Zhang was very happy to take away the children’s toy.)

NORM: semantically and syntactically normal. SEM: semantic violation. SEM + SYN: double violations. Examples are given in Chinese, with English literal glosses and translations below. The critical words are in bold.

As the SEM and SEM + SYN conditions are primarily concerned with linguistic manipulations, three experts of Chinese linguistics verified the semantic and syntactic constraints of these sentences with perfect consistency, that is, all of them agreed that all the SEM sentences contained semantic violations, all the SEM + SYN sentences contained both syntactic and semantic violations, and that all the Norm and filler sentences were both semantically and syntactically acceptable. In addition, the NORM sentences as well as the filler sentences were randomly mixed with the SEM and the SEM + SYN sentences, and a group of Chinese native speakers (*N* = 10) who did not participate in the formal experiment were asked to determine whether these sentences were natural and thus acceptable to them or not by Yes/No responses through a questionnaire (without time limits). All NORM and filler sentences (100% correctly responded as “Yes”) could be well distinguished from the SEM and the SEM + SYN sentences (100% correctly responded as “No”) for each participant. Therefore, the results showed robust consistency across the participants and explanatory power regarding the validity of the materials.

The critical words in the experimental sentences were primarily selected from the glossary of HSK level-5 or below, with which L2 learners were asked to familiarize themselves before the experiment. The frequency and the number of strokes of the critical words were matched across three conditions (frequency information retrieved from Cai and Brysbaert, 2010; see Table 2). One-way ANOVA showed that there were no significant differences regarding the number of strokes and word frequency of the critical words among different conditions, *F*s(2, 117) < 2.18, *p*s > 0.05. In particular, the critical words did not appear at the end of the sentences to avoid ERP contamination from the “wrap-up effect” at sentence-final positions (Hagoort, 2003). Except for the critical words, the sentential contexts were identical across conditions. Specifically, all experimental sentences used “*小张*” (Zhang) as the subject to avoid possible expectations caused by the sentence subject. Additionally, the frequency of the nouns following the functional word “*的*” (de) in the NORM condition was balanced in terms of the argument roles as “tool” (e.g., “knife”) or “agent” (e.g., “child”). An additional single word reading task was conducted to provide a baseline for the ERP correlates underlying the critical words. These materials contained only nouns and verbs (45 words for each category), which were identical to the critical words adopted in the experimental sentences.

**Table 2:** The number of strokes and word frequency of critical words in different conditions.

Condition	Number of the strokes	Frequency
NORM	13.800 ± 4.697	3.228 ± 0.646
SEM	14.200 ± 3.451	3.006 ± 0.539
SEM + SYN	15.150 ± 4.240	3.256 ± 0.571

### 2.3. Procedure

Word reading and sentence comprehension tasks were conducted for both groups, while behavioral performance and EEG signals were recorded simultaneously. L2 speakers received the list of critical words 2 days before the experiments and were asked to consolidate the memory of these words. In addition, they were required to review these words again prior to the formal experiment until they reported total familiarity with the meaning and usage of the words.

In the formal experiment, participants were seated approximately 60–70 cm from the computer monitor in a fully shielded laboratory. Participants performed the word reading task before the sentence comprehension session. In the word reading task, each trial began with the presentation of a red fixation at the center of the screen for approximately 300 ms, followed by a blank screen for 200 ms, the critical word for 500 ms, and then a 1,000 ms blank screen. The participants were asked to familiarize themselves with the words again in this session. To ensure the participants’ engagement, they were informed that their real-time mental activities would be monitored by the device. To note, first, the objective of asking participants (especially for the L2 learners) to familiarize themselves with the critical words before the formal test was to ensure that the processing differences of the critical words between the conditions during sentence reading could not be ascribed to the mere familiarity effects. Second, more critically, for each condition, a critical word could appear in all the tasks of word familiarization, word reading, and of sentence comprehension (see below). Therefore, the potential preview effect (i.e., effect of seeing the “critical words” multiple times before the formal study including both single-word reading and sentence comprehension tasks) could be canceled out in the comparison between different conditions if there were any for each stage.

The sentence comprehension task consisted of three blocks with 54, 54, and 52 sentences in each block. The order of the three blocks was randomized and there was a two-minute interval between every two consecutive blocks. In each block, three conditions of experimental sentences and filler sentences were presented in a pseudorandom order, such that the sentences of the same condition would not appear in more than three times consecutively. Each trial began with a red fixation cross at the center of the screen for approximately 300 ms, followed by sentences presented word by word, e.g., “小张 | 拿着 | 切 | 水果 | 的 | 小刀 | 过来了。” [Zhang | was taking | cut | fruit | de (functional word) | knife | came over.]. In particular, double-character words were presented for 500 ms, while single-character words lasted for 400 ms. Each word was followed by a 300 ms blank screen, while a Chinese period (“。”) indicated the end of the sentence. After the sentence disappeared, the participants were required to judge whether the sentence was acceptable within 3,000 ms, which was followed by a blank screen lasting for 1,000 ms. The whole experiment took approximately 1.5 h for each participant.

### 2.4. Data recordings and analyses

Electroencephalogram (EEG) was recorded using a 32-channel (Ag-AgCl) NeuroScan system (NeuroScan Inc.) following the 10–20 system convention. For online recordings, the reference electrode was placed at the right mastoid (M2). Vertical electrooculogram (VEOG) was recorded from electrodes placed above and below the left eye, while horizontal EOG (HEOG) was recorded from electrodes at the outer cantus of each eye. The data were digitized at 1 kHz and amplified with a bandpass filter of 0.05–100 Hz. The impedance of each channel was maintained below 5 kΩ.

Off-line signal processing was carried out using Scan software (NeuroScan Inc.). The reference was converted to the averaged voltages of the bilateral mastoids (M1 and M2). EEG data were first adjusted by eliminating artifacts using the DC method and regression analysis and then segmented into 1,000 ms epochs, including a 200-ms prestimulus baseline and an 800-ms poststimulus. Eye blinks and other artifacts exceeding ±100 μV were rejected. ERPs were averaged across conditions for both word reading and sentence comprehension tasks and then filtered again with a low bandpass filter of 30 Hz (24 dB).

**Figure 1:**
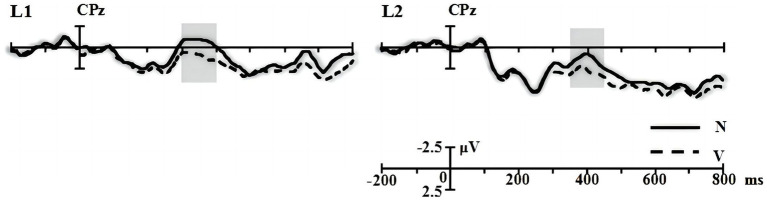
The averaged waveforms elicited by nouns (N) and verbs (V) in the word reading task. The grey rectangle marked the greatest difference between the two categories.

**Figure 2:**
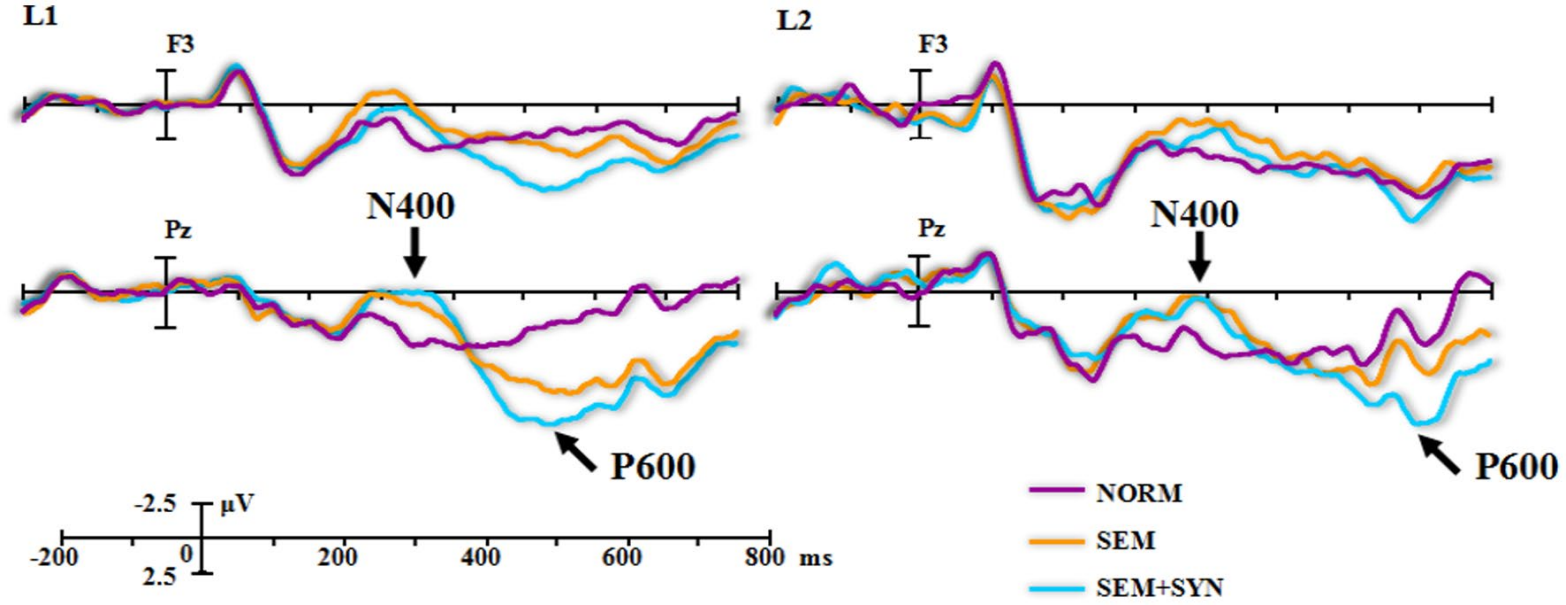
The original brain waveforms locked to the critical words embedded in the experimental sentences.

**Figure 3:**
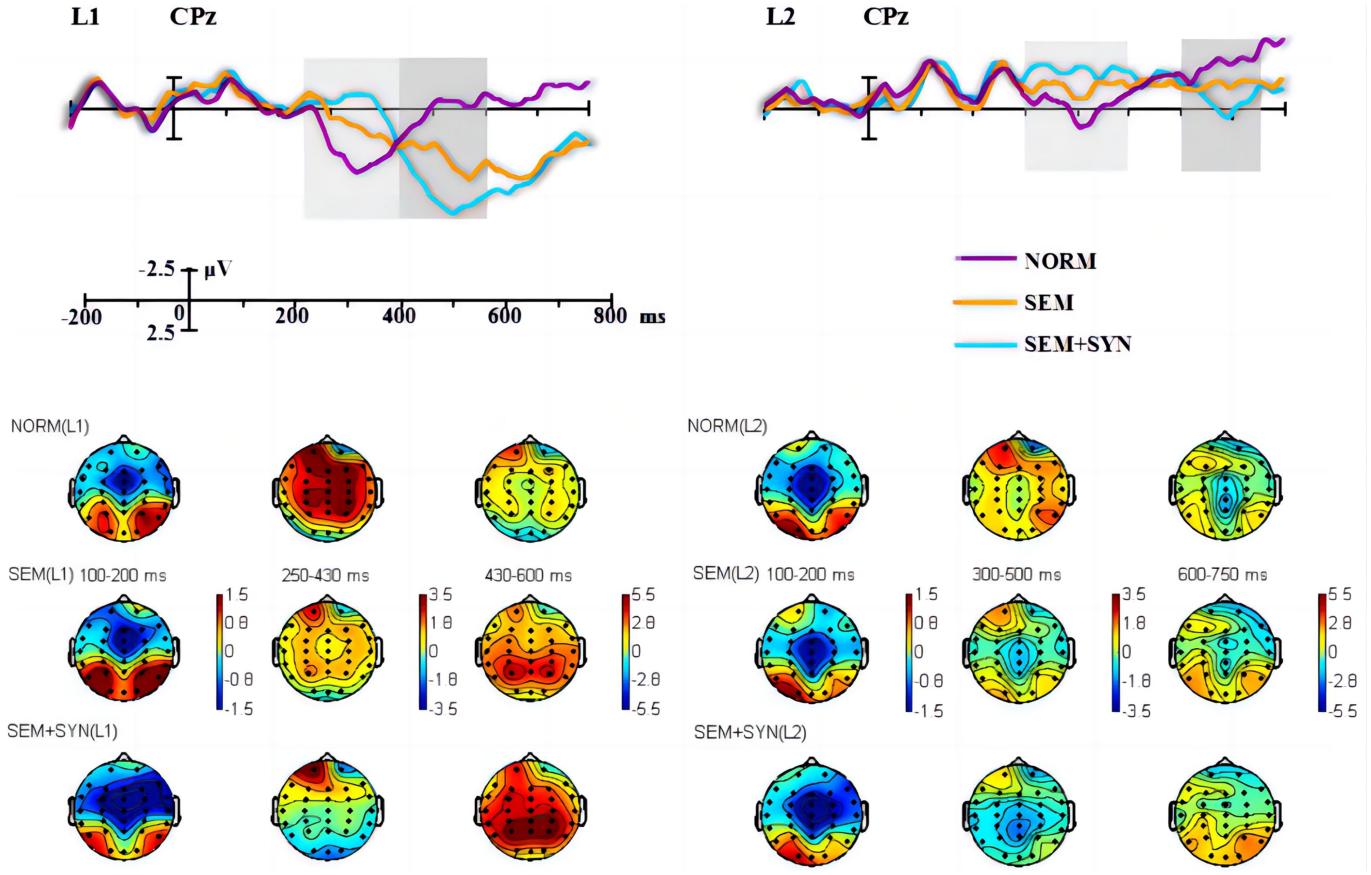
The grand-averaged difference waveforms and topographies. Light and dark gray rectangles marked the most obvious differences across conditions in the middle and late time-windows, respectively.

**Figure 4:**
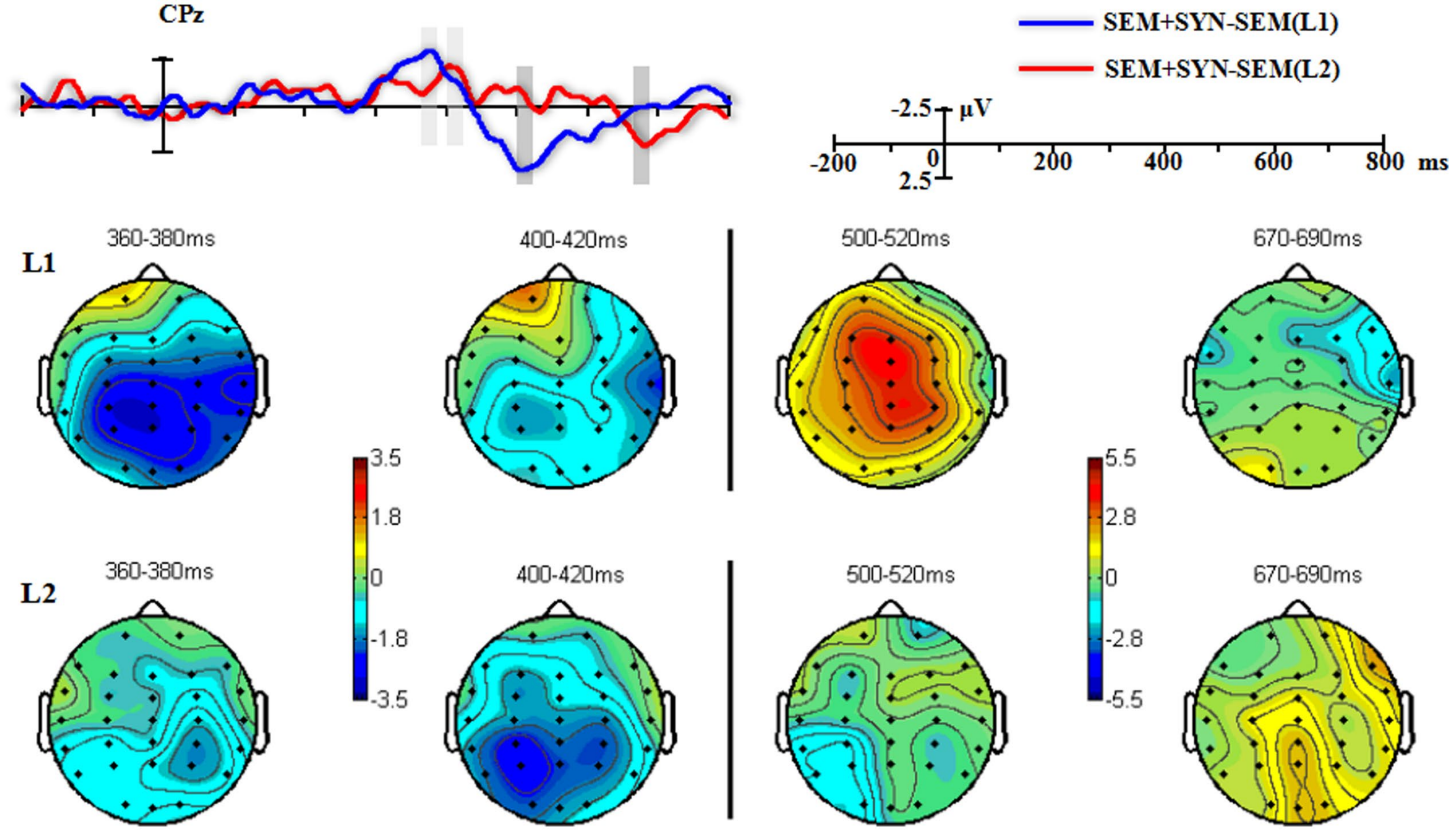
The grand-averaged difference waveforms and topographies of “SEM + SYN - SEM.” Light and dark gray rectangles marked the most obvious differences across conditions in the middle and late time-windows, respectively.

The ERP components induced by nouns and verbs in the word reading task (see Figure 1) were subtracted from the ERP components induced by the corresponding critical words in different types of experimental sentences (see Figure 2), so the word reading results were not included in the later statistical analyses (see Figure 1). As the L1 and L2 groups showed differing time windows of ERP components (i.e., N400 and P600) in the sentence comprehension task (see Figure 3), we first conducted statistical analysis within each group and then compared the components of interest between groups. For the within-group analyses, based on the visual inspection of brain topographies (see Figure 3) and previous studies (e.g., Yu and Zhang, 2008; Zhang et al., 2010; Wang et al., 2015), we selected three time windows of 100–200 ms, 250–430 ms, and 430–600 ms for the Chinese L1 group and 100–200 ms, 300–500 ms and 600–750 ms for L2 learners. They were used to denote ELAN, N400 (or larger negativities), and P600 (or late positivities), respectively. In light of existing ERP studies, we defined 7 regions of interest (ROIs) for statistical analyses: midline (Fz, FCz, Cz, CPz, and Pz), left anterior (F3, FC3, F7, and FT7), left central (C3 and T7), left posterior (CP3, P3, TP7, and P7), right anterior (F4, FC4, F8, and FT8), right central (C4 and T8), and right posterior (CP4, P4, TP9, and P8). As such, we first conducted a 3-way ANOVA on ELAN data at the midline sites, on which the L1 and L2 groups shared the same time window, with the condition (NORM, SEM, and SYN + SEM), electrode (Fz, FCz, Cz, CPz, and Pz), and group (L1 and L2) as factors. For the lateral regions, a 3-way ANOVA was conducted with condition, ROIs (left anterior, left central, left posterior, right anterior, right central, and right posterior), and group as factors. Then, identical analyses were conducted on N400 and P600 data, except that the group factor was removed due to the differing time windows across the two groups. Furthermore, we took a narrow-window approach (e.g., Sanmiguel et al., 2013; Ghani et al., 2020) by selecting the data including 10 ms before and 10 ms after the N400/P600 peaks since the wider time window mentioned above might overwhelm the subtle differences across conditions (as shown in Figure 4). Furthermore, representative electrodes were also selected based on the topography (Figure 4) to conduct the independent-sample t test between the experimental condition (SEM + SYN - SEM) and “baseline (0).” Importantly, narrow-window analysis provided a viable tool for group comparisons regarding N400 and P600 effects. Furthermore, *post hoc* power analysis using G*power (Faul et al., 2007) was performed for *main* results so as to evaluate the overall statistic power of the present study.

## 3. Results

### 3.1. Behavioral results

**Figure 5:**
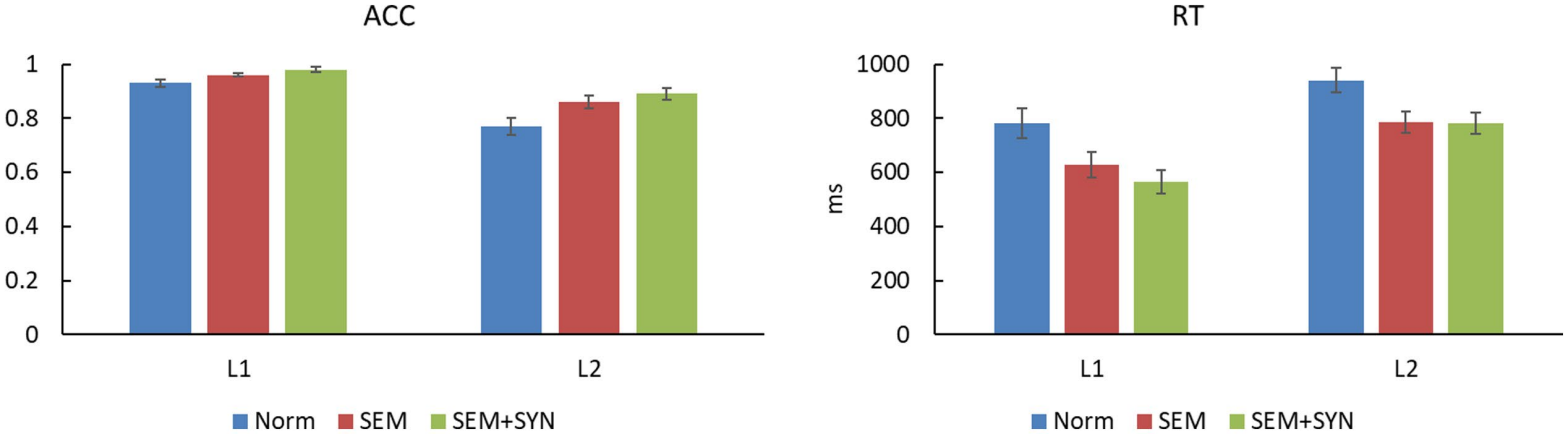
The ACCs and RTs of behavioral results (error bars indicate standard errors).

The accuracy rate (ACC)^4^ and reaction time (RT) data of L1 and L2 speakers across the three conditions are shown in Table 3 and Figure 5. ACC data showed a significant main effect of condition, *F*(2, 76) = 17.91, *p* < 0.0005, *η_p_^2^* = 0.320, *power* = 0.97. Pairwise comparisons with Bonferroni corrections revealed that the ACC of the SEM + SYN condition was significantly higher than that of the SEM and NORM conditions (*p*s < 0.005), while the ACC of the SEM condition was significantly higher than that of the NORM condition (*p* < 0.005). The main effect of group was significant, *F*(1, 38) = 26.77, *p* < 0.001, *η_p_^2^* = 0.413, *power* > 0.99, such that L1 speakers obtained significantly higher accuracy than L2 learners. The interaction between the two factors was not significant, *F*(2, 76) = 2.30, *p* = 0.132. In terms of RT data, the main effect of condition was significant [*F*(2, 76) = 58.50, *p* < 0.0005, *η_p_^2^* = 0.606, *power* > 0.99]. Pairwise comparison showed that the RT of SEM + SYN was significantly shorter than that of SEM and NORM *(p*s < 0.01), while the responses to SEM were significantly faster than those to NORM (*p* < 0.001). The main effect of group was significant [*F*(1, 38) = 8.59, *p* < 0.01, *η_p_^2^* = 0.184, *power* = 0.83]. The RT of L1 Chinese speakers was significantly shorter than that of L2 Chinese speakers. The interaction between the two factors above was not significant [*F*(2, 76) = 1.58, *p* = 0.212].

**Table 3:** Behavioral performance of the two groups.

Group	Condition	ACC	RT (ms)
L1 (*N* = 20)	NORM	0.93 ± 0.06	780.31 ± 246.73
	SEM	0.96 ± 0.03	627.84 ± 212.15
	SEM + SYN	0.98 ± 0.04	564.46 ± 195.70
L2 (*N* = 20)	NORM	0.77 ± 0.14	941.04 ± 206.59
	SEM	0.86 ± 0.11	786.36 ± 175.19
	SEM + SYN	0.89 ± 0.10	781.09 ± 180.70

### 3.2. ERP results

As seen from the original waveforms of the three conditions across the two groups (Figure 2), the SEM + SYN condition elicited N400- and P600-like components, while ELAN was not detected at the frontal sites (F3 as the representative electrode) for both groups. In addition, single word reading results (Figure 1) showed that nouns induced more negative waves than verbs in the 300–500 ms time window for both groups. We then subtracted the waveforms of word categories from the original waveforms of the critical words embedded in sentential contexts (Figure 3) to better evaluate the ERP modulation of syntactic and semantic processing.

#### 3.2.1 ELAN results

ELAN effects were examined at the frontal sites in the time window of 100–200 ms. At the midline sites, there was no significant effect for condition [*F*(2, 76) = 1.40, *p* = 0.253]. At the lateral sites, the main effect of condition was also not significant [*F*(2, 76) = 2.79, *p* = 0.081]. Thus, for both groups, the SEM + SYN condition did not induce an ELAN effect in the early time window.

#### 3.2.2 N400 results

**Figure 6:**
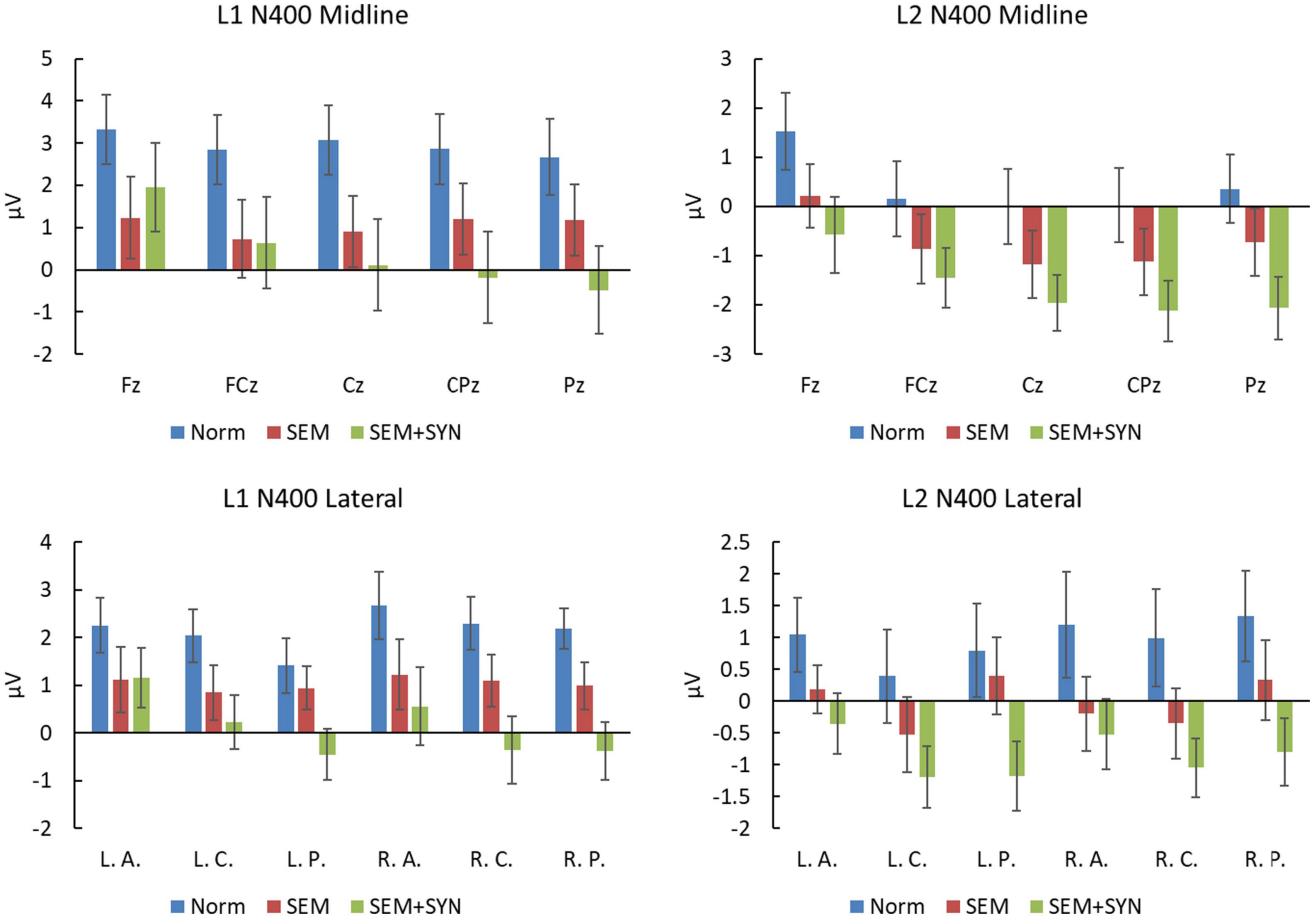
Amplitudes at midline electrodes and lateral ROIs in the N400 time window (250–430 ms for L1 and 300–500 ms for L2). L. A.: left anterior; L. C.: left central; L. P.: left posterior; R. A.: right anterior; R. C.: right central; R. P.: right posterior.

As shown in Figure 6, L1 data revealed a significant condition effect at both the midline and lateral sites [*F*s(2, 38) > 11.90, *p*s <0.001, *η_p_^2^*s > 0.385, *power* > 0.99] in the N400 time window (250–430 ms). Pairwise comparisons showed that SEM + SYN (midline: 0.407 ± 4.537 μV; lateral: 0.126 ± 2.406 μV) and SEM (midline: 1.049 ± 3.653 μV; lateral: 1.035 ± 2.140 μV) elicited more negative waves than NORM (midline: 2.953 ± 3.470 μV; lateral: 2.142 ± 2.101 μV) at both the midline and lateral sites (*p*s < 0.05), while no difference was detected between SEM + SYN and SEM. At the midline sites for L1 speakers, the main effect of electrode was also significant [*F*(4, 76) = 4.57, *p* < 0.05, *η_p_^2^* = 0.194, *power* > 0.99] such that FCz (1.402 ± 2.168 μV) yielded more negative waves than Fz (2.168 ± 4.054 μV; *p* < 0.05). In general, the negativities in this time window showed a central-posterior distribution in the midline region. The interaction between electrode and condition was not significant [*F*(8, 152) =0.73, *p* = 0.524]. For the lateral regions, the ROI effect of L1 speakers was not significant [*F*(5, 95) = 1.00, *p* = 0.379], while its interaction with condition was significant [*F*(10, 190) = 2.78, *p* < 0.05, *η_p_^2^* = 0.251, *power* > 0.99]. Simple effect analyses showed comparable ERP patterns across all lateral ROIs as midline sites [*F*s(2, 38) ≥ 6.30, *p*s < 0.005].

In the time window of 300–500 ms, L2 data manifested similar patterns as L1 data. There was a significant condition effect for both midline [*F*(2, 38) = 6.36, *p* < 0.05, *η_p_^2^* = 0.251, *power* > 0.99; see Figure 6] and lateral sites [*F*(2, 38) = 7.34, *p* < 0.01, *η_p_^2^* = 0.279, *power* > 0.99] such that the difference between the SEM (midline: −0.737 ± 2.688 μV; lateral: −0.026 ± 1.987 μV) and SEM + SYN (midline: −1.624 ± 2.574 μV; lateral: −0.851 ± 1.854 μV) conditions was not significant, while the SEM and SEM + SYN conditions yielded more negative waves than the NORM condition (midline: 0.411 ± 3.129 μV; lateral: 0.961 ± 2.771 μV; *p*s < 0.05). The main effect of electrode was also significant at midline sites [*F*(4, 76) = 4.57, *p* < 0.05, *η_p_^2^* = 0.194, *power* > 0.99]. FCz (−0.720 ± 2.639 μV) yielded more negative waves than Fz (0.388 ± 2.764 μV; *p* < 0.05). In general, the negative waves in this time window showed a centro-posterior distribution in the midline region. The interaction between distribution and condition was not significant [*F*(8, 152) = 0.73, *p* = 0.524]. No other significant main effect or interation was detected at lateral ROIs.

To eliminate semantic contaminations from the double violations, we analyzed the difference wave between SEM + SYN and SEM in light of a narrower window of 20 ms (i.e., 10 ms prior to the peak and 10 ms after the peak, see Figure 4). For L1 speakers, there were wide negativities in the posterior sites in the time window of 360–380 ms. Repeated-measures ANOVA with the experimental condition (SEM + SYN - SEM vs. baseline “0”) and electrode (CPz, Pz, CP3, CP4, P3, P4) as factors revealed a significant effect of the experimental condition (SEM + SYN – SEM: −2.621 ± 3.760 μV), *F*(1, 19) = 9.72, *p* < 0.01, *η_p_^2^* = 0.338, *power* > 0.99, while no other significant main effect or interaction was detected [*F*s(5, 95) ≤0.78, *p*s > 0.05]. Double violations elicited significantly greater negativities, which were widely distributed at bilateral centro-parietal regions. For L2 learners, SEM + SYN elicited more obvious negativities than SEM in 400–420 ms at centro-parietal sites (Figure 4). A similar repeated-measures ANOVA was conducted on representative sites (Cz, CPz, Pz, C3, C4, CP3, CP4, P3, P4). There was a significant effect of experimental condition [*F*(1, 19) = 19.62, *p* < 0.0005, *η_p_^2^* = 0.506, *power* > 0.99] such that SEM + SYN was associated with significantly greater N400 than SEM (SEM + SYN – SEM: *M* = −2.204 μV, *SD* = 2.937 μV). No other significant main effect or interaction was identified.

**Figure 7:**
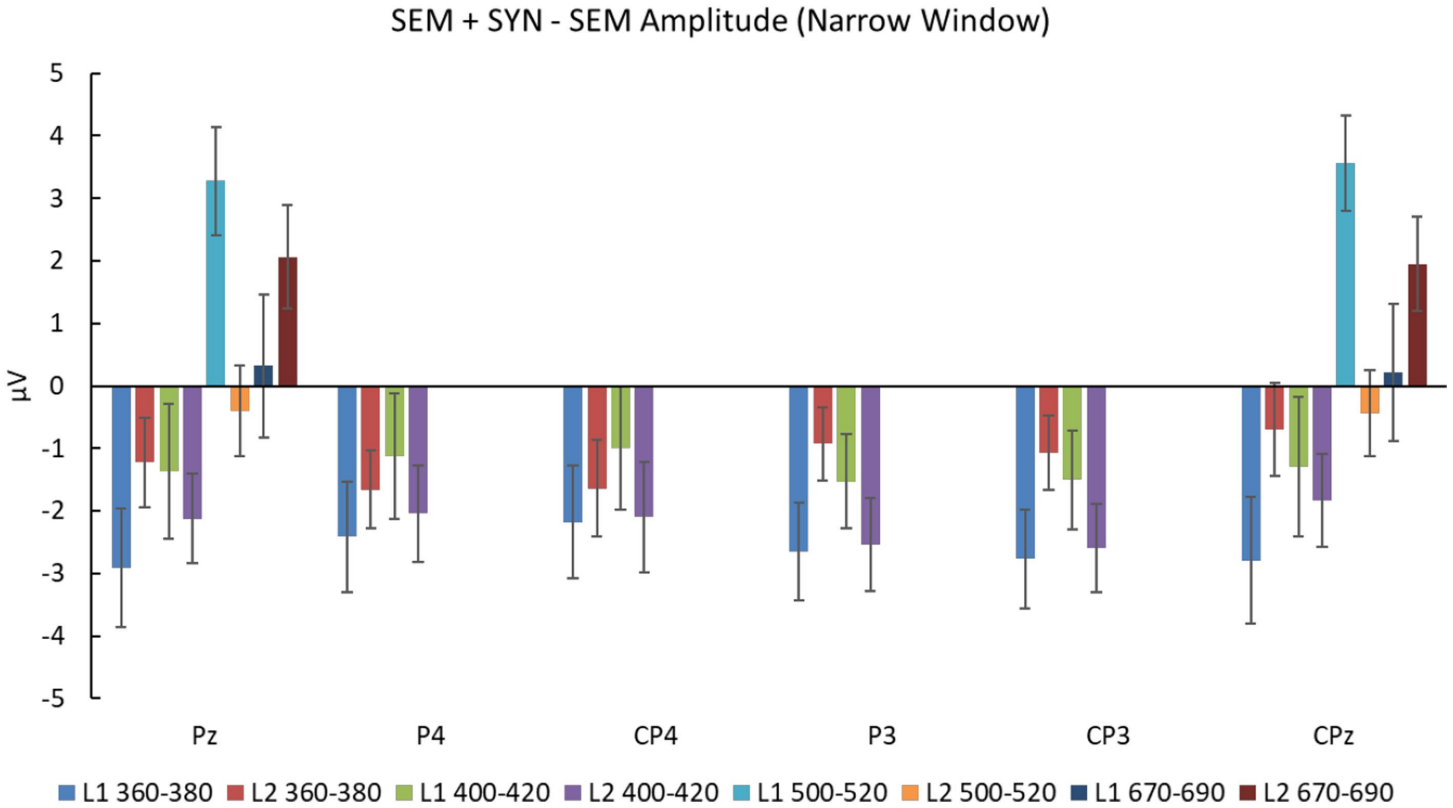
SEM + SYN – SEM amplitudes at selected electrodes for the chosen time window (360–380 and 400–420 ms for N400 narrow window analyses at Pz, P4, CP4, P3, CP3, and CPz; 500–520 and 670–690 ms for P600 narrow window analyses at Pz and CPz).

**Figure 8:**
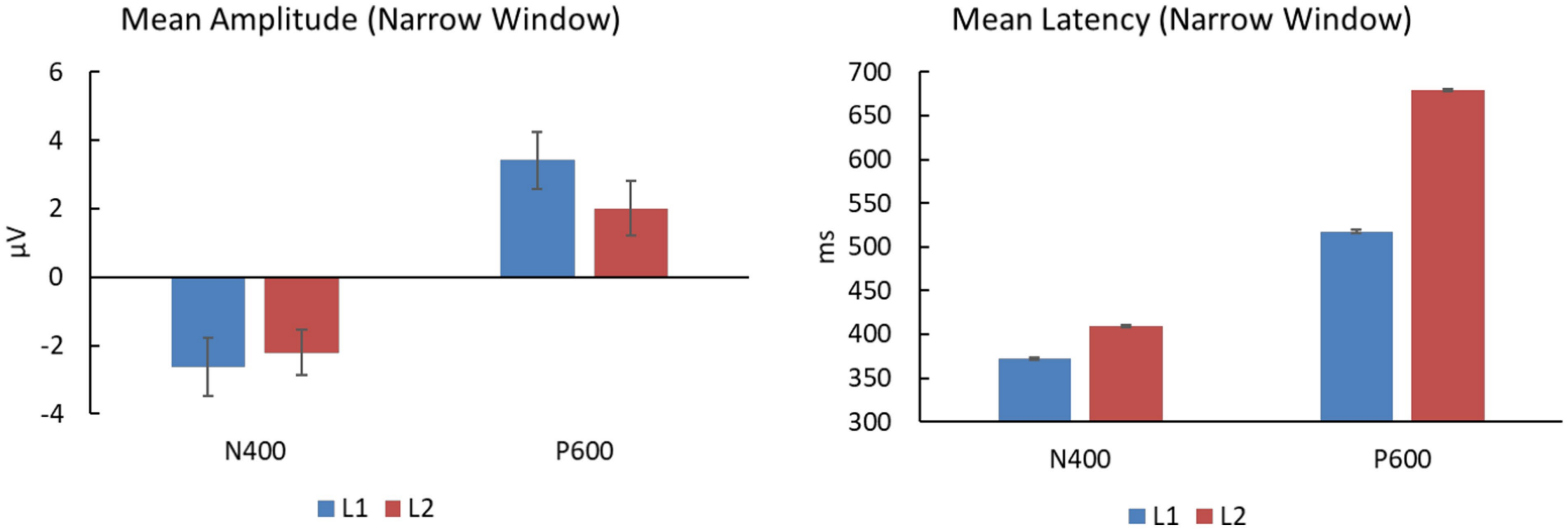
Mean amplitudes and latency averaged from selected electrodes for narrow-window analyses (360–380 ms for L1 and 400–420 ms for L2 in N400 time window; 500–520 ms for L1 and 670–690 ms for L2 in P600 time window).

We further analyzed the group difference in N400 difference waves (SEM + SYN - SEM) by conducting a 3-way repeated-measures ANOVA with electrodes (CPz, Pz, CP3, CP4, P3, P4), time window (360–380 ms, 400–420 ms), and group (L1 vs. L2) as factors (Figure 7). Only a significant interaction between time window and group was detected, *F*(1, 38) = 11.78, *p* < 0.005, *η_p_^2^* = 0.237, *power* > 0.99, such that the N400 effect between two time windows was significant for both groups [*F*s(1, 38) ≥ 4.40, *p*s <0.05]. In addition, N400 peak results averaged from the six centro-parietal sites (360–380 ms for L1 and 400–420 ms for L2) showed a significant group effect on peak latency [*t*(38) = −18.27, *p* < 0.0005; see Figure 8], while there was no significant effect on peak amplitudes across groups [*t*(38) = −0.39, *p* = 0.698; see Figure 8]. In particular, the latency of L1 speakers (372.592 ± 6.138 ms) was significantly earlier than that of L2 learners (408.975 ± 6.453 ms).

#### 3.2.3 P600 results

**Figure 9:**
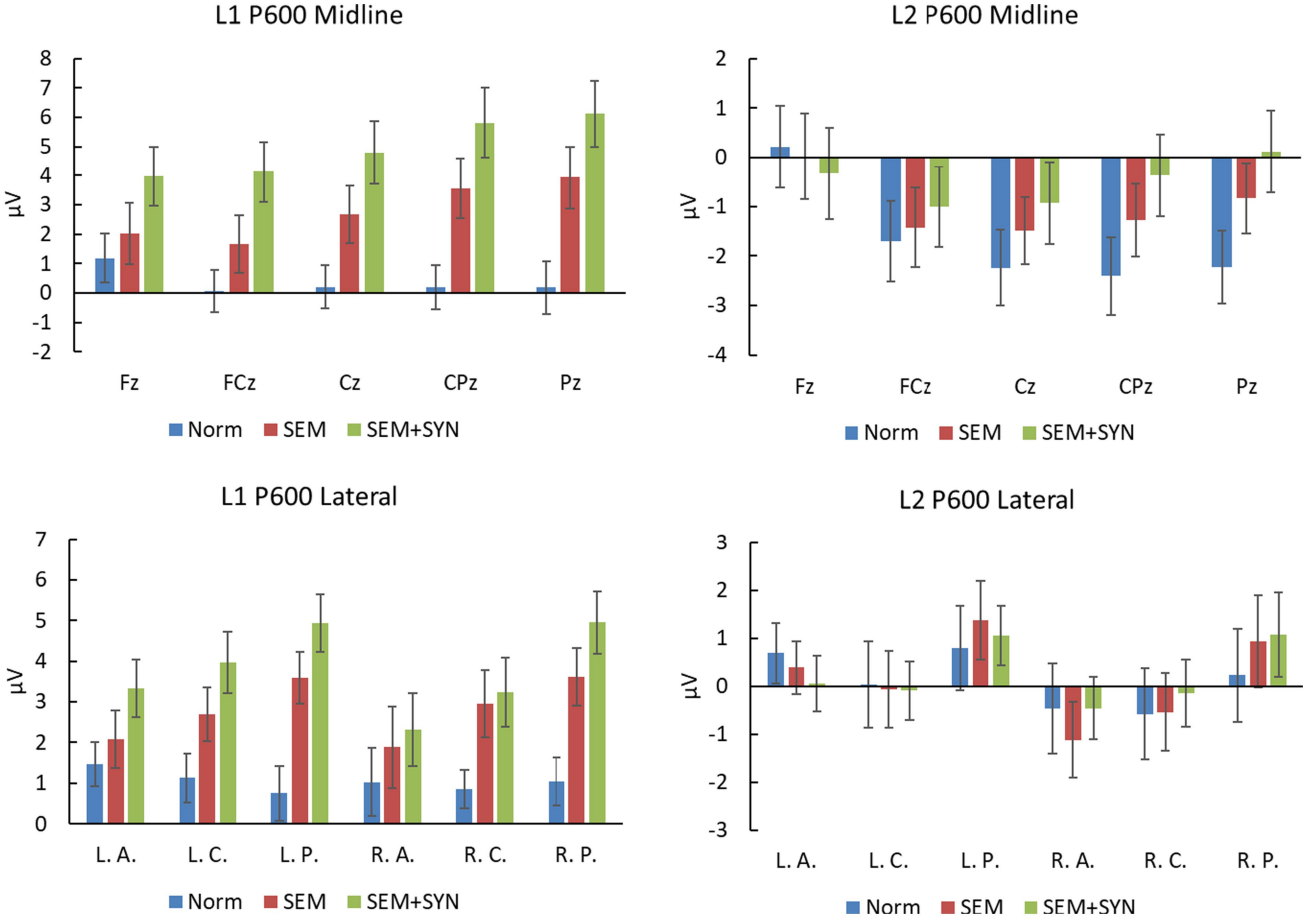
Amplitudes at midline electrodes and lateral ROIs in P600 time window (430–600 ms for L1 and 600–750 ms for L2). L. A.: left anterior; L. C.: left central; L. P.: left posterior; R. A.: right anterior; R. C.: right central; R. P.: right posterior.

At midline sites, the 2-way ANOVA yielded a significant main condition effect [*F*(2, 38) = 18.05, *p* < 0.0005, *η_p_^2^* = 0.487, *power* > 0.99; see Figure 9]. The following comparisons showed that SEM + SYN (4.964 ± 4.462 μV) induced more positive waves than SEM (2.768 ± 4.200 μV), while SEM induced more positive waves than NORM (0.364 ± 3.067 μV; *p*s <0.05). The main effect of electrode was not significant [*F*(4, 76) = 1.98, *p* = 0.170]. The interaction between condition and electrode was significant [*F*(8, 152) = 11.18, *p* < 0.001, *η_p_^2^* = 0.371, *power* > 0.99]. Simple effect analyses showed that the condition effect was significant at all electrodes [*F*s(2, 38) ≥ 3.72, *p*s <0.05]. At lateral ROIs, the main effect of condition was significant [*F*(2, 38) = 11.79, *p* <0.0005, *η_p_^2^* = 0.383, *power* > 0.99] such that SEM + SYN (3.787 ± 2.896 μV) and SEM (2.800 ± 2.856 μV) elicited more positive waves than NORM (1.046 ± 2.049 μV; *p*s < 0.01), while no difference between the two violation conditions was detected (*p* = 0.305). The main effect of ROI was not significant [*F*(5, 95) = 1.77, *p* = 0.191], but its interaction with condition was significant [*F*(10, 190) = 5.36, *p* <0.005, *η_p_^2^* = 0.220, *power* > 0.99]. In particular, the condition difference was significant at all ROIs [*F*s(2, 38) ≥ 4.36, *p*s <0.05], except at the right anterior region [*F*(2, 38) = 1.36, *p* = 0.268].

As seen in Figure 9, the P600 results in the L2 group manifested a distinct pattern from L1 speakers. At midline sites, the main effects of condition [*F*(2, 38) = 1.55, *p* = 0.230] and electrode [*F*(4, 76) = 3.09, *p* = 0.077] were not significant, while the interaction between them was significant [*F*(8, 152) = 6.23, *p* < 0.005, *η_p_^2^* = 0.247, *power* > 0.99]. Simple effect analyses showed that condition effects were significant at Pz and CPz [*F*s(2, 38) ≥ 4.175, *p*s < 0.05] such that SEM + SYN and SEM elicited more positive waves than NORM (*p*s > 0.05), while no difference between the two violation conditions was detected (*p* = 0.305). At lateral ROIs, neither a significant main effect nor a significant interaction was identified.

Narrow-window analysis results based on difference waves in the L1 group (500–520 ms) showed wide positivities in bilateral centro-parietal regions (Figure 4). Repeated-measures ANOVA with the experimental condition (SEM + SYN - SEM vs. baseline “0”) and electrode (CPz, Pz, CP3, CP4, P3, P4) as factors revealed a significant effect of the experimental condition, *F*(1, 19) = 17.45, *p* < 0.01, *η_p_^2^* = 0.479, *power* > 0.99, such that SEM + SYN elicited significantly greater P600 than SEM (SEM + SYN – SEM: 3.417 ± 3.674 μV). There was no other significant main effect or interaction. For L2 learners, identical analysis was performed on CPz and Pz in the time window of 670–690 ms. There was a significant main effect of experimental condition, *F*(1, 19) = 6.23, *p* < 0.05, *η_p_^2^* = 0.247, *power* > 0.99. SEM + SYN elicited greater P600 than SEM (SEM + SYN – SEM: *M* = 2.008 ± 3.597 μV). The main effect of electrode and the interaction were not significant, *F*s(2, 38) < 0.22, *p*s > 0.05.

As shown in Figure 4, the L1 and L2 groups produced obvious positivities associated with SEM + SYN – SEM at the time windows of 500–520 ms and 670–690 ms, respectively. We therefore examined the group difference at representative sites CPz and Pz (Figure 7). Repeated-measures ANOVA revealed a significant interaction only between time window and group [*F*(1, 38) = 22.164, *p* < 0.0005, *η_p_^2^* = 0.368, *power* > 0.99]. Specifically, the group effect was significant only in the time window of 500–520 ms, *F*(1, 38) = 12.45, *p* < 0.001, while the time-window effect was significant for both groups, *F*s(1, 38) ≥ 4.66, *p*s < 0.05. Averaged results on P600 peaks across two representative sites showed comparable peak values for the two groups [*t*(38) = 1.23, *p* = 0.228], while the L1 group (517.425 ± 7.281 ms) showed significantly shorter peak latency than the L2 group (679.125 ± 7.527 ms), *t*(38) = −69.06, *p* < 0.0005 (Figure 8).

In addition, to examine the lateralization of ERP components, we subsequently averaged the lateral electrodes on left (F3, FC3, F7, FT7, C3, T7, CP3, P3, TP7, and P7) and right (F4, FC4, F8, FT8, C4, T8, CP4, P4, TP9, and P8) hemisphere and conducted a 2-way ANOVA on N400 and P600 data for L1 and L2 groups respectively, with condition (NORM, SEM, and SEM + SYN) and hemisphere (left and right) as factors. The results were shown in Supplementary material.

## 4. Discussion

Drawing on behavioral and ERP techniques, the current study investigated the relationship between syntactic and semantic processing when reading Chinese complex sentences with relative clauses center-embedded among L1 speakers and highly proficient L2 learners whose native language was Korean. Our findings showed that L1 speakers and L2 learners manifested a consistent behavioral and electrophysiological pattern of highly interactive syntactic and semantic processing during Chinese complex sentence reading.

The behavioral performance showed that SEM + SYN yielded higher ACC than SEM and NORM, while NORM was associated with the lowest accuracy. Likewise, SEM + SYN showed the fastest reactions, followed by SYN and NORM. Importantly, the behavioral patterns of the L1 and L2 groups were generally consistent. In particular, SEM + SYN involved both syntactic and semantic violations, which made error detection easier and faster than typical sentences. Although SEM involved semantic violations, its syntactic information remained correct, which led to relatively better recognition performance than double violations. In contrast, L1 and L2 speakers needed to make use of all the information available until all contents of the sentence were integrated when reading the semantically and syntactically normal sentences, which resulted in the longest reaction time and the lowest accuracy.

Furthermore, the ERP results provided more nuanced insights into the interaction between syntactic and semantic information in L1 and L2 Chinese complex sentence reading. The first important ERP finding from the current study was that L1 and L2 Chinese complex sentences with center-embedded relative clauses did not elicit the ELAN effect in the frontal sites from the double violations, while ELAN is an index of initial syntactic processing. This finding was consistent with the ERP patterns obtained from the double violations in simpler Chinese syntactic structures including BA, BEI, and SVO constructions (Yu and Zhang, 2008; Zhang et al., 2010; Wang S. et al., 2013; Wang et al., 2015; Zeng et al., 2020), where ELAN was also not identified. Those evidence could collectively suggest that syntactic processing is not the prerequisite for semantic processing in Chinese sentence comprehension regardless of structural complexities. In particular, ELAN has been recognized as an important index of temporal priority for syntactic processing and automaticity for local structure building (Friederici et al., 1993; Hahne and Friederici, 2002; Hagoort and Indefrey, 2014). However, ELAN is susceptible to various experimental manipulations. Steinhauer and Drury (2012) concluded that ELAN might be associated with a higher distribution probability of some stimuli (e.g., affix), the asymmetry of the precritical-word context with unchanged critical words, the “spillover effect,” and the “offset effect.” For instance, Fromont et al. (2016) created the French word category violation within a constant sentential context by using the homophone of definite articles “le/la/les” (equivalent to “the” in English) and accusative attachments “le/la/les” (equivalent to “him or her/them” in English), in which ELAN was not detected. Furthermore, Fromont et al. (2020) attempted to mitigate the interference from critical word alternation by changing presentence contexts and the type of critical words in the experimental sentences. Nevertheless, no ELAN effect was identified. Likewise, studies on Chinese sentences failed to find ELAN when keeping the sentential context unchanged (e.g., Yu and Zhang, 2008; Zhang et al., 2010, Experiment I, 2013; Wang S. et al., 2013; Wang et al., 2015; Yang et al., 2015). Furthermore, word category violation is deemed as syntactic violation from the linguistic perspective, while in Chinese, a word category violation is always accompanied with the semantic violation, and thus it can serve as the double-violation condition, which is valid for examining the interplay between syntactic and semantic processing in both alphabetic languages and Chinese. In light of the established rationales (e.g., Ye et al., 2006; Zhang et al., 2010, 2013), we could attribute the absence of ELAN to the null temporal and functional priority of syntax over semantics in Chinese, thus in support of the notion that in Chinese syntactic processing (esp., reflected by the word category combinations) is highly interactive with semantic processing as Chinese is assumed to depend on meanings heavily.

In addition, although ELAN was mostly identified from auditory experiments, the possibility cannot be ruled out that ELAN could be elicited from visual presentations (Gunter and Friederici, 1999; Friederici, 2017). For example, Dikker et al. (2009) noted that early syntactic processing might involve visual perception and analysis, which would facilitate the time course of syntactic processing. As the current study presented all sentences visually, the absence of ELAN in the current study might not relate to the input modality of language stimuli (but see Limitation for further discussion).

Even though ELAN was absent for both groups, L1 speakers and L2 learners manifested greater negativities in the N400 time window and greater positivities in the P600 time window in bilateral central-posterior sites when recognizing double violations than pure semantic violations. In particular, the priority of syntactic information processing could also be reflected by the block effect from syntactic violation on subsequent semantic processing (Steinhauer and Drury, 2012). Consistent with existing studies on Chinese simple structures (Yu and Zhang, 2008; Wang S. et al., 2013; Zhang et al., 2013; Zeng et al., 2020), the current study detected greater N400 from double violations than from the NORM condition, which is not supportive of a block effect. Meanwhile, this N400 was widely distributed at the central-posterior sites on the human scalp, which is in line with these related ERP studies. However, previous studies pointed out that the blocked semantic N400 might be affected by task demands and material properties. For instance, Hahne and Friederici (2002, Experiment II) found that N400 was elicited from double-violation sentences when the participants were engaged in a semantic judgment task. The authors therefore believed that semantic processing requires more cognitive control and is not as automatic as syntactic processing. Likewise, Zhang et al. (2010) admitted that the task of their Experiment I, which required the participants to answer questions related to the semantics of the experimental sentences, might also involve a bias toward semantic processing, thus resulting in larger N400 for double-violation sentences. However, they also identified significantly larger negativities associated with the double violations in experiment II, which employed an overall acceptability judgment task that was neutral with regard to syntactic and semantic processing.

In addition to task demands, N400 and the block effect could be impacted by the accessibility order of syntactic and semantic information in the experimental materials (Hahne and Friederici, 2002; van den Brink and Hagoort, 2004). In Hahne and Friederici’s (2002) double-violation sentence “*Das Turschloß wurde im gegessen*.,” syntactic violation was created by word category violation in “*im gegessen*” locally, while semantic violation was realized by the long-distance violation between “*Das Turschloß*” and “*gegessen*.” It is thus debatable that the asymmetry of the violation distance may induce ELAN and further cause a block effect. In other words, the extraction of the subject N “*Das Turschloß*” from working memory and the establishment of the relationship between the subject and the predicate verb “*gegessen*” were obviously slower than the immediate collocation between adjacent words (i.e., “*im gegessen*”). As such, the first violation the participants encountered was the violation of word category. However, Zhang et al. (2010) employed a similar manipulation as Hahne and Friederici (2002) in Experiment II, where ELAN and block effects were still not found in the asymmetry of violation distance.

To resolve the confounding factors mentioned above on the N400 findings, the current study employed an overall acceptability judgment task (Zhang et al., 2010) to prevent participants’ potential semantic bias. Importantly, our study focused on Chinese complex sentences where the disagreements between critical words and their collocation took place at the same distance for both the SEM and SEM + SYN conditions. As such, word syntactic information and semantic information could be accessed at the same time. Previous studies used Chinese simple sentences including “BA” and “BEI” structures as experimental materials, where syntactic processing may be weakened due to the relatively local syntactic violation. However, the syntactic complexity of our complex sentences was relatively higher, which could better reflect the role of word category information in long-distance dependency processing (see also Gibson, 1998). Furthermore, in our study, the critical words were placed in the middle of experimental sentences instead of at the end. This operation could effectively eliminate the wrap-up effect on ERP signals (Hahne and Friederici, 2002; Hagoort, 2003). Collectively, we can further verify that Chinese syntactic and semantic information are processed in parallel rather than in a serial manner (Kuperberg, 2007; Zhang et al., 2013; Fromont et al., 2020).

As expected, we observed that SEM + SYN induced significantly larger negativities than SEM in the N400 time window for both L1 and L2 speakers. As N400 conventionally indexes a process of semantic violation detection, our findings suggested an interactive pattern of syntactic and semantic violations in this time window. The consequences of a semantic violation on the N400 amplitude were boosted by an additional syntactic violation, while there was no boost of syntactic violation on P600 amplitude by additional semantic violation, thus manifesting an asymmetric pattern between semantic and syntactic processing (Hagoort, 2003). As such, we interpret the enhanced N400 in the SEM + SYN condition as additional difficulty in semantic integration from syntactic violation.

In line with previous findings (Wang S. et al., 2013; Wang et al., 2015), our study identified late positivities in the P600 time-window at bilateral centro-parietal sites for both SEM + SYN and SEM conditions. In addition, the enhanced P600 elicited by SEM + SYN compared with SEM tended to manifest an overlap between syntactic and semantic violations. Importantly, this pattern was consistent for both L1 speakers and L2 learners. P600 has been associated with the integration (Kaan et al., 2000; Friederici, 2011) and restoration (Hagoort et al., 1993; Friederici et al., 2002a; Kaan and Swaab, 2003) of various types of sentence information. In contrast, Zhang et al. (2013) found that double violations of verb transitivity and semantics did not induce the late positive component in the P600 time window compared with normal sentences. The authors thus interpreted the P600 as a sensitivity to the degree of sentence abnormality. Frisch et al. (2004) found that syntactic violation represented by word category violation could block the processing of argument structure, while the simple argument structure violation was set by verb transitivity violation. As such, verb transitivity violation may be lower than word category violation with regard to the degree of syntactic violation. Similarly, syntactic violation and double violations induced comparable P600 patterns among French L1/L2 speakers (Fromont et al., 2020) and Chinese L1 speakers (Lu, 2015; Li, 2016). However, as these studies used simple sentences where syntactic violations were always contaminated by semantic violations to some extent, the interplay between syntactic and semantic information in the P600 time window remains to be elucidated. Our study also detected a P600 associated with pure semantic violation in both groups. Kuperberg (2007) held that when semantic information was abnormal, a semantic memory-based stream could generate semantic illusion, which could decrease N400 amplitudes and merge the lexical entries into a combinatorial stream. This combination could enable a semantic (re)analysis and further cause a P600 component. In addition, P600 caused by semantic violation might reflect a combinatorial mechanism of semantics and syntax from a more general sense. Therefore, for both L1 and L2 speakers in our study, the observed P600 related to SEM + SYN and SEM might implicate an integral index of final repair and integration of semantics and syntax in complex sentence comprehension. One contribution of our study with regard to this issue is that we extended the P600 findings to Chinese L2 learners reading complex sentences. Even though their L1 and L2 exhibit linguistic differences, highly proficient L2 learners can still present similar patterns for syntactic processing as native speakers of the target language from the absence of ELAN to greater N400 and P600 associated with SEM + SYN than NORM. Taken together, our L2 findings are consistent with Friederici et al.’s (2002b) notion that highly proficient L2 learners can develop native-like processing strategies. For both groups, syntax does not present a temporal and functional priority over semantics, and there is an intensive interaction between syntactic and semantic information in the N400 time window such that double violations are associated with enhanced negativities due to accumulated semantic and syntactic information (Hagoort, 2003). In the later time window of P600, positivities could reflect participants’ repair and integrity of the complex structures.

However, although L2 learners and L1 speakers had similar processing patterns shown by ERP, the difference waves’ latency of “SEM + SYN - SEM” of L2 learners was longer than that of L1 speakers. This result was consistent with Yang (2012), who investigated the differences between L1 Chinese speakers and highly proficient German-speaking Chinese L2 speakers in processing the Mandarin “BEI” structure. According to the between-group analysis results as well as the visualization in Figure 4, L2 learners showed a significant delay of the N400 and P600 latencies. Thus, L2 speakers were still slower than L1 speakers in detecting, repairing, and integrating syntactic and semantic violations when reading Chinese complex sentences.

Specifically, the unified competition model (MacWhinney, 1997, 2005) holds that L2 learners employ the cognitive resources and processing strategies from their L1 to address L1-L2 shared structures. However, in regard to a marked contrast between L1 and L2, it remains unknown how L2 learners from different learning stages process the complex L1 structure. Our results revealed that highly proficient L2 learners developed parallel patterns to L1 speakers when processing center-embedded Chinese relative clauses, while the only difference was shown in the longer latency. The difference between L1 and L2 implies a difference in degree, not in kind, thus supporting a unified competition account. Although existing studies have established the role of L2 proficiency (Bowden et al., 2013; Yu and Dong, 2018; Jin et al., 2019), the similar processing pattern between L1 speakers and L2 learners indicates that given adequate language exposure, L2 learners can effectively suppress the influence of their mother tongue (i.e., L1) and show native-like sentence processing. In particular, the interaction between syntactic and semantic information processing in reading Chinese sentences plays a similar role for both L1 speakers and highly proficient L2 learners. Nevertheless, we found a difference in the ERP latencies, which might suggest the additional time that L2 learners need to process, repair, and integrate syntactic and semantic information. In light of the declarative/procedural (DP) model (Ullman, 2015), in both L1 and L2, the knowledge of syntax should initially be learned in declarative memory. In parallel, procedural memory should also gradually develop. After sufficient exposure to the language, procedural memory-based syntactic processing should take precedence over analogous declarative knowledge, resulting in increasing automatization of syntactic processing, which provides the opportunity for L2 learners to develop native-like automatic processing. However, Ullman (2015) also noted that even after years of exposure, adult L2 learners might not attain the degree of proceduralization of their syntax as L1 or early L2 learners because the ability of procedural memory gradually fades with increasing learners’ age. In other words, there seems to be an unbridgeable gap in the automaticity of sentence processing between L1 speakers and late L2 learners. However, based on the current results, we propose that the nature of this gap might be quantitative rather than qualitative.

## 5. Conclusion

In light of ERP techniques, the current study examined how syntactic category information and semantics interact with each other regarding the time course of reading Chinese complex sentences among L1 speakers and highly proficient L2 learners. Our results revealed that double violations of semantics and syntax did not elicit an ELAN effect for either group. In addition, double violations evoked enhanced N400 and P600 compared with normal sentences, thus exhibiting a consistent biphasic waveform pattern. These findings indicate a highly interactive relationship between syntactic and semantic information during Chinese complex sentence reading and suggest that syntax does not manifest a temporal and functional priority, which could relate to the typological specialties of the Chinese language system. Importantly, Chinese L2 learners with a morphologically rich language background could effectively suppress the influence from their L1 and show a similar ERP pattern to native Chinese speakers. Our findings further suggest that the syntax-first pattern in L2 might be limited to specific languages such that there might exist an interaction between L2 proficiency and language typology. Languages with differing morphological diversity might exhibit different electrophysiological patterns regarding the interplay between syntactic and semantic processes.

## 6. Limitation

The current study has several limitations warranting discussion.

First, participants’ gender differences were not well controlled in each group. Even though both L1 and L2 groups shared a similar gender ratio (L1: male:female = 6/17; L2: male:female = 7/16) in the present study (see also Wang S. et al., 2013: 6 to 15; Wang et al., 2015: 4 to 17 for a similar ratio), making the results comparable between the groups and the studies, whether and to what extend the gender factor would modulate the relationship between semantic and syntactic processing during Chinese complex sentence reading still await to be explored.

Second, the working memory capacities were comparable across the two groups (L1 vs. L2), and for the materials, as the critical word position in the sentence was identical across all the conditions (Norm, SEM, and SEM+, SYN), WM variations (if any) should not be confounded with our results. Nevertheless, we did not further examine whether and how the individual working memory differences might modulate the sentence processing, which is a valuable research question for further investigations.

Third, we only aimed to investigate whether relatively-high-proficiency Chinese L2 learners with a distinct language background from Chinese could process the syntactic and semantic information as Chinese L1 speakers, Thus, the potential modulation effect of L2 proficiency was not in the focus of the present study. Nevertheless, future studies may include more languages and various language proficiency levels to address these issues in a more systematic fashion.

Last, although ELAN was mostly identified from auditory experiments, evidence that it could appear in reading studies (i.e., in the visual modality) was also reported (e.g., Neville et al., 1991; Dikker et al., 2009; Wang S. et al., 2013). As such, we attributed the absence of ELAN to the absence of initial syntactic processing, the rationale of which is also in line with existing studies in Chinese (Yu and Zhang, 2008; Zhang et al., 2010; Wang S. et al., 2013; Wang et al., 2015; Zeng et al., 2020), rather than the mere modality effect. However, conducting the present experiment in the auditory domain is expected in the near future so as to evaluate whether the ELAN effect would be amplified in the auditory modality.

## Data availability statement

The raw data supporting the conclusions of this article will be made available by the authors, without undue reservation.

## Ethics statement

The studies involving human participants were reviewed and approved by Ethics Committee of Beijing Normal University. The patients/participants provided their written informed consent to participate in this study.

## Author contributions

LC and LF: came up with the original idea of this study. LC: conducted the ERP experiment, and analyzed the data. LC, MY, and FG: completed the first draft of this manuscript, which was further revised by ZF, PW, and LF. All authors participated in the discussion of the results and prepared the revised version of the manuscript for submission.

## Funding

This work was funded by National Social Science Fund of China (22CYY017).

## Conflict of interest

The authors declare that the research was conducted in the absence of any commercial or financial relationships that could be construed as a potential conflict of interest.

## Publisher’s note

All claims expressed in this article are solely those of the authors and do not necessarily represent those of their affiliated organizations, or those of the publisher, the editors and the reviewers. Any product that may be evaluated in this article, or claim that may be made by its manufacturer, is not guaranteed or endorsed by the publisher.

## Footnotes

^1^BA structure is constructed by a subject (NP1) followed by the preposition BA and the object (i.e., NP2), and finally a VP. The preposition BA forces the default word order in Chinese [i.e., subject–verb–object (SVO)] into subject–BA–object–verb to emphasize the meaning of disposal. For example, *设计师把布料裁了* [literal glosses: the stylist BA the cloth cut le (*functional word*); translation: the stylist cut the cloth] (Jin, 1997; Ye et al., 2006; Zhang et al., 2010).

^2^BEI structure [subject (NP1) + BEI + object (NP2) + VP] in Chinese is used as the passive voice structure, which is similar to the passive voice structure in English [subject (NP1) + be + VP-ed + by + object (NP2)]. E.g., *自行车被小偷偷走了* [literal glosses: the bike BEI a thief stolen away le (*functional word*); translation: the bike was stolen away by a thief] (Wang S. et al., 2013; Wang et al., 2015).

^3^Chinese QING structure consists of the following items in sequence: QING + NP1 + VP + NP2, which is similar to “ask + NP1 + to + VP + NP2” in English. Qing structure is frequently used to express imperative mood in Chinese, and it sounds polite. E.g., *请你打扫这个房间* [literal glosses: QING you clean up this room; translation: please clean up this room] (Yang et al., 2021).

^4^ACC reflects the correct acceptance of NORM condition and the correct rejection of SEM and SEM + SYN conditions.
