# Supplementary material for: Mandarin Chinese L1 and L2 complex sentence reading reveals a consistent electrophysiological pattern of highly interactive syntactic and semantic processing: An ERP study
Source: Front Psychol. 2023 Apr 19;14:1143062. doi: 10.3389/fpsyg.2023.1143062 (PMC10155869; doi:10.3389/fpsyg.2023.1143062)
Supplement: Supplementary file 1 [file Data_Sheet_1.pdf]

Supplementary Material

1 Supplementary Analyses of Averaged ROIs

To further examine the lateralization of ERP components and make the results clearer, we subsequently averaged the lateral ROIs on the left (F3, FC3, F7, FT7, C3, T7, CP3, P3, TP7, and P7) and right (F4, FC4, F8, FT8, C4, T8, CP4, P4, TP9, and P8) hemispheres and conducted a 2-way ANOVA on N400 and P600 data for L1 and L2 groups, respectively, with condition (NORM, SEM, and SEM + SYN) and hemisphere (left and right) as factors.

N400 results

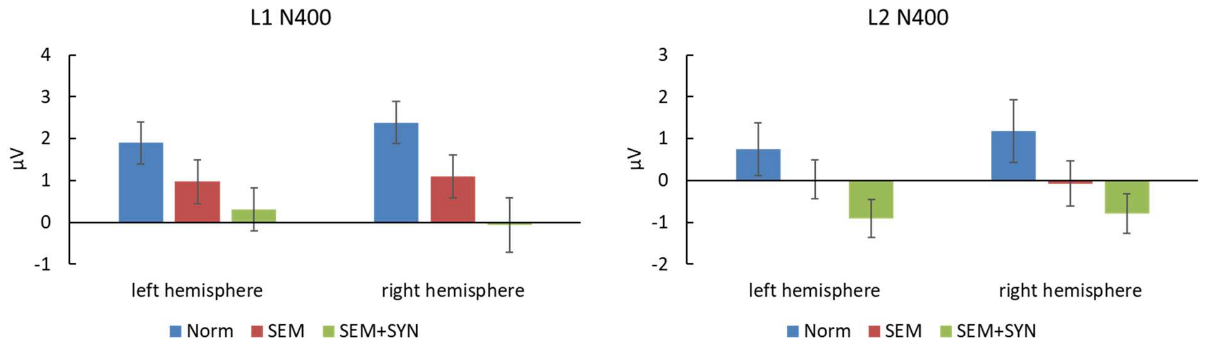

Supplementary Figure 1. Amplitudes at averaged ROIs in the left and right hemispheres in N400 time window (250-420 ms time window for L1 and 300-500 ms time window for L2).

L1 data revealed a significant condition effect [$F(2, 38) = 17.20$, $p < .001$, $\eta_p^2 = .475$]. A *post hoc* comparative analysis further showed that SEM + SYN ($M = .126 \pm 2.406$ μV) and SEM ($M = 1.035 \pm 2.140$ μV) elicited more negative waves than NORM ($M = 2.142 \pm 2.101$ μV; *p*s <.05), while no difference was detected between SEM + SYN and SEM ($p = .091$). The main effect of hemisphere [$F(1, 19) = .60$, $p = .809$] and the interaction between hemisphere and condition [$F(2, 38) = 2.41$, $p = .104$] were not significant (Supplementary Figure 1).

L2 data manifested similar patterns as L1 data in N400 time window. A main effect of condition was detected [$F(2, 38) = 7.34$, $p = .009$, $\eta_p^2 = .279$]. A *post hoc* comparative analysis further showed that SEM + SYN ($M = -.851 \pm 1.854$ μV) and SEM ($M = -.027 \pm 1.987$ μV) elicited more negative waves than NORM ($M = .961 \pm 2.771$ μV; *p*s <.05), while no difference was detected between SEM + SYN and SEM ($p = .334$). The main effect of hemisphere [$F(1, 19) = .12$, $p = .734$] and the interaction between hemisphere and condition [$F(2, 38) = .76$, $p = .433$] were not significant (Supplementary Figure 1).

P600 results

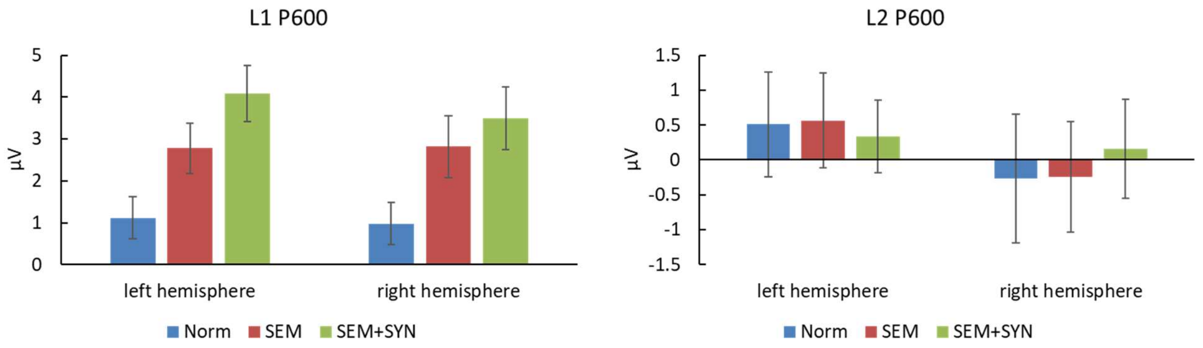

Supplementary Figure 2. Amplitudes at averaged ROIs in the left and right hemisphere in P600 time window (430-600 ms time window for L1 and 600-750 ms time window for L2).

L1's P600 results yielded a significant main effect of condition [$F(2, 38) = 11.79$, p <.001, η_p^2 =.383]. A *post hoc* comparative analysis further showed that SEM + SYN ($M = 3.787 \pm 2.986$ µV) and SEM ($M = 2.800 \pm 2.856$ µV) elicited more positive waves than NORM ($M = 1.046 \pm 2.049$ µV; *ps* <.01), while no difference was detected between SEM + SYN and SEM (p =.305). The main effect of hemisphere [$F(1, 19)$ =.39, p =.539] and the interaction between hemisphere and condition [$F(2, 38)$ = 1.03, p =.366] were not significant (Supplementary Figure 2).

The P600 results in the L2 group manifested a distinct pattern from L1 speakers such that neither significant main effects [$Fs(2, 38)$ <.93, *ps* >.348] nor a significant interaction [$F(2, 38) = 1.16$, p =.324] was detected (Supplementary Figure 2).

The supplementary analyses manifested only main condition effect for all time windows except P600 window for L2, such that SEM + SYN and SEM elicited more positive waves than NORM, while no difference was detected between SEM + SYN and SEM. No lateralization or interaction was detected in the supplementary analyses. These results are consistent with analyses on separated lateral ROIs.

2 Supplementary Tables

The means and standard deviations of amplitudes as well as latencies of every time window and every electrode or ROI for both L1 and L2 groups are given below in form of tables.

Supplementary Table 1. Amplitudes (μV) at midline electrodes in the ELAN time window (100-200 ms).

Group	Condition	Fz	FCz	Cz	CPz	Pz
	NORM	-1.462 ± 3.282	-1.730 ± 2.955	-1.248 ± 2.877	-0.607 ± 2.903	-0.023 ± 2.855
L1 (*N* = 20)	SEM	-1.565 ± 3.393	-1.744 ± 2.971	-1.248 ± 2.623	-0.434 ± 2.628	0.212 ± 2.890
	SEM + SYN	-1.409 ± 2.430	-2.048 ± 2.427	-1.795 ± 2.800	-1.065 ± 2.929	-0.550 ± 2.992
	NORM	-1.144 ± 3.446	-1.908 ± 3.005	-1.979 ± 2.682	-1.456 ± 2.525	-0.915 ± 2.464
L2 (*N* = 20)	SEM	-0.664 ± 2.695	-1.379 ± 2.438	-1.593 ± 2.323	-1.144 ± 2.481	-0.814 ± 2.388
	SEM + SYN	-1.651 ± 3.973	-2.462 ± 3.286	-2.386 ± 2.762	-1.706 ± 2.849	-1.137 ± 2.737

Supplementary Table 2. Amplitudes (μV) at lateral ROIs in the ELAN time window (100-200 ms). L. A.: left anterior; L. C.: left central; L. P.: left posterior; R. A.: right anterior; R. C.: right central; R. P.: right posterior.

Group	Condition	L. A.	L. C.	L. P.	R. A.	R. C.	R. P.
	NORM	-0.687 ± 2.002	-0.552 ± 1.701	0.396 ± 1.478	-0.915 ± 2.384	-0.637 ± 2.172	0.609 ± 2.310
L1 (*N* = 20)	SEM	-0.571 ± 2.336	-0.374 ± 2.055	0.796 ± 1.706	-0.739 ± 1.704	-0.259 ± 1.355	1.053 ± 2.394
	SEM + SYN	-0.928 ± 1.640	-0.852 ± 1.281	0.370 ± 1.394	-1.562 ± 1.698	-1.213 ± 1.864	0.326 ± 2.558
	NORM	-0.614 ± 2.311	-0.616 ± 2.435	0.313 ± 2.352	-0.613 ± 2.621	-0.631 ± 2.404	0.131 ± 2.358
L2 (*N* = 20)	SEM	-0.310 ± 1.587	-0.538 ± 1.968	0.427 ± 2.012	-0.459 ± 1.822	-0.628 ± 1.628	0.141 ± 1.567
	SEM + SYN	-0.697 ± 2.466	-0.790 ± 2.106	0.062 ± 2.394	-1.267 ± 2.143	-1.307 ± 1.976	-0.397 ± 2.220

Supplementary Table 3. Amplitudes (μV) at midline electrodes in the N400 time window (250-430 ms for L1 and 300-500 ms for L2).

Group	Condition	Fz	FCz	Cz	CPz	Pz
	NORM	3.324 ± 3.707	2.839 ± 3.666	3.067 ± 3.693	2.863 ± 3.723	2.672 ± 4.040
L1 (*N* = 20)	SEM	1.227 ± 4.348	0.729 ± 4.120	0.908 ± 3.795	1.199 ± 3.760	1.184 ± 3.763
	SEM + SYN	1.952 ± 4.710	0.639 ± 4.846	0.110 ± 4.843	-0.189 ± 4.838	-0.479 ± 4.628
	NORM	1.525 ± 3.490	0.153 ± 3.392	0.001 ± 3.399	0.027 ± 3.404	0.351 ± 3.109
L2 (*N* = 20)	SEM	0.214 ± 2.852	-0.865 ± 3.154	-1.175 ± 3.044	-1.125 ± 3.037	-0.735 ± 3.065
	SEM + SYN	-0.574 ± 3.462	-1.449 ± 2.734	-1.960 ± 2.511	-2.120 ± 2.757	-2.067 ± 2.826

Supplementary Table 4. Amplitudes (μV) at lateral ROIs in the N400 time window (250-430 ms for L1 and 300-500 ms for L2). L. A.: left anterior; L. C.: left central; L. P.: left posterior; R. A.: right anterior; R. C.: right central; R. P.: right posterior.

Group	Condition	L. A.	L. C.	L. P.	R. A.	R. C.	R. P.
	NORM	2.250 ± 2.569	2.036 ± 2.493	1.414 ± 2.559	2.671 ± 3.200	2.298 ± 2.498	2.185 ± 1.927
L1 (*N* = 20)	SEM	1.117 ± 3.114	0.849 ± 2.579	0.942 ± 2.033	1.227 ± 3.291	1.090 ± 2.458	0.985 ± 2.193
	SEM + SYN	1.158 ± 2.811	0.227 ± 2.536	-0.452 ± 2.392	0.560 ± 3.688	-0.363 ± 3.149	-0.376 ± 2.729
	NORM	1.044 ± 2.632	0.390 ± 3.313	0.798 ± 3.278	1.198 ± 3.746	0.995 ± 3.435	1.339 ± 3.200
L2 (*N* = 20)	SEM	-0.191 ± 1.696	0.529 ± 2.661	-0.398 ± 2.717	0.199 ± 2.590	0.350 ± 2.471	-0.331 ± 2.805
	SEM + SYN	-0.360 ± 2.139	-1.195 ± 2.195	-1.179 ± 2.449	-0.520 ± 2.455	-1.050 ± 2.064	-0.801 ± 2.397

Supplementary Table 5. Amplitudes (μV) at midline electrodes in the P600 time window (430-600 ms for L1 and 600-750 ms for L2).

Group	Condition	Fz	FCz	Cz	CPz	Pz
	NORM	1.185 ± 3.752	0.058 ± 3.253	0.206 ± 3.243	0.187 ± 3.386	0.184 ± 3.986
L1 (*N* = 20)	SEM	2.016 ± 4.718	1.662 ± 4.429	2.657 ± 4.382	3.566 ± 4.574	3.937 ± 4.725
	SEM + SYN	-1.565 ± 3.393	-1.744 ± 2.971	-1.248 ± 2.623	-0.434 ± 2.628	0.212 ± 2.890
	NORM	0.210 ± 3.701	-1.698 ± 3.649	-2.241 ± 3.414	-2.403 ± 3.490	-2.225 ± 3.265
L2 (*N* = 20)	SEM	0.014 ± 3.905	-1.428 ± 3.596	-1.483 ± 3.035	-1.276 ± 3.326	-0.831 ± 3.239
	SEM + SYN	-0.323 ± 4.135	-1.006 ± 3.654	-0.926 ± 3.74	-0.37 ± 3.725	0.114 ± 3.751

Supplementary Table 6. Amplitudes (μV) at lateral ROIs in the P600 time window (430-600 ms for L1 and 600-750 ms for L2). L. A.: left anterior; L. C.: left central; L. P.: left posterior; R. A.: right anterior; R. C.: right central; R. P.: right posterior.

Group	Condition	L. A.	L. C.	L. P.	R. A.	R. C.	R. P.
	NORM	1.465 ± 2.389	1.129 ± 2.710	0.748 ± 3.038	1.030 ± 3.735	0.863 ± 2.111	1.041 ± 2.635
L1 (*N* = 20)	SEM	2.079 ± 3.183	2.685 ± 2.955	3.585 ± 2.824	1.880 ± 4.470	2.948 ± 3.713	3.620 ± 3.160
	SEM + SYN	3.325 ± 3.188	3.972 ± 3.375	4.935 ± 3.203	2.307 ± 4.013	3.238 ± 3.830	4.946 ± 3.449
	NORM	0.690 ± 2.806	0.040 ± 4.045	0.793 ± 3.937	-0.460 ± 4.213	-0.578 ± 4.251	0.228 ± 4.367
L2 (*N* = 20)	SEM	0.387 ± 2.483	-0.064 ± 3.579	1.376 ± 3.647	-1.123 ± 3.531	-0.537 ± 3.611	0.941 ± 4.280
	SEM + SYN	0.048 ± 2.595	-0.093 ± 2.704	1.062 ± 2.784	-0.462 ± 2.926	-0.140 ± 3.135	1.079 ± 3.955

Supplementary Table 7. SEM + SYN – SEM amplitudes (μV) at selected electrodes for the chosen time window (360-380 ms and 400-420 ms for N400 narrow window analyses at Pz, P4, CP4, P3, CP3, and CPz; 500-520 ms and 670-690 ms for P600 narrow window analyses at Pz and CPz).

Time Window	Group	Pz	P4	CP4	P3	CP3	CPz
360-380 ms	L1	-2.914 ± 4.219	-2.418 ± 3.922	-2.183 ± 4.036	-2.650 ± 3.465	-2.765 ± 3.524	-2.797 ± 4.529
	L2	-1.227 ± 3.187	-1.656 ± 2.818	-1.641 ± 3.472	-0.925 ± 2.621	-1.063 ± 2.662	-0.699 ± 3.320
400-420 ms	L1	-1.375 ± 4.820	-1.127 ± 4.526	-1.001 ± 4.409	-1.526 ± 3.353	-1.498 ± 3.539	-1.290 ± 4.968
	L2	-2.122 ± 3.197	-2.043 ± 3.439	-2.097 ± 3.942	-2.538 ± 3.300	-2.590 ± 3.158	-1.836 ± 3.319
500-520 ms	L1	3.275 ± 3.978	-	-	-	-	3.559 ± 3.508
	L2	-0.396 ± 3.312	-	-	-	-	-0.435 ± 3.144
670-690 ms	L1	0.318 ± 5.285	-	-	-	-	0.212 ± 5.007
	L2	2.063 ± 3.781	-	-	-	-	1.952 ± 3.485

Supplementary Table 8. Latencies (ms) from selected electrodes for narrow-window analyses on chosen time window.

Group	360-380 ms	400-420 ms	500-520 ms	670-690 ms
L1	372.592 ± 6.138	406.742 ± 5.941	517.425 ± 7.281	677.725 ± 6.478
L2	370.700 ± 7.310	408.975 ± 6.453	523.375 ± 6.898	679.125 ± 7.527
